# Rrp5 Binding at Multiple Sites Coordinates Pre-rRNA Processing and Assembly

**DOI:** 10.1016/j.molcel.2013.10.017

**Published:** 2013-12-12

**Authors:** Simon Lebaron, Åsa Segerstolpe, Sarah L. French, Tatiana Dudnakova, Flavia de lima Alves, Sander Granneman, Juri Rappsilber, Ann L. Beyer, Lars Wieslander, David Tollervey

**Affiliations:** 1 Wellcome Trust Centre for Cell Biology, University of Edinburgh, Michael Swann Building, Kings Buildings, Mayfield Road, Edinburgh EH9 3JR, Scotland; 2 Department of Molecular Biosciences, WGI, Stockholm University, 106 91 Stockholm, Sweden; 3 Department of Microbiology, Immunology and Cancer Biology, University of Virginia Health System, Charlottesville, VA 22908-0734, USA; 4 SynthSys, JR Waddington Building, Kings Buildings, Mayfield Road, Edinburgh EH9 3JR, Scotland

## Abstract

In vivo UV crosslinking identified numerous preribosomal RNA (pre-rRNA) binding sites for the large, highly conserved ribosome synthesis factor Rrp5. Intramolecular complementation has shown that the C-terminal domain (CTD) of Rrp5 is required for pre-rRNA cleavage at sites A0–A2 on the pathway of 18S rRNA synthesis, whereas the N-terminal domain (NTD) is required for A3 cleavage on the pathway of 5.8S/25S rRNA synthesis. The CTD was crosslinked to sequences flanking A2 and to the snoRNAs U3, U14, snR30, and snR10, which are required for cleavage at A0–A2. The NTD was crosslinked to sequences flanking A3 and to the RNA component of ribonuclease MRP, which cleaves site A3. Rrp5 could also be directly crosslinked to several large structural proteins and nucleoside triphosphatases. A key role in coordinating preribosomal assembly and processing was confirmed by chromatin spreads. Following depletion of Rrp5, cotranscriptional cleavage was lost and preribosome compaction greatly reduced.

## Highlights

• Rrp5 binds multiple dispersed sites in the pre-rRNA
• The NTD and CTD of Rrp5 each bind adjacent to sites of cleavages that require them
• Rrp5 directly binds large, structural proteins and NTPases
• Rrp5 is required for preribosome compaction

## Graphical Abstract

**Figure:**
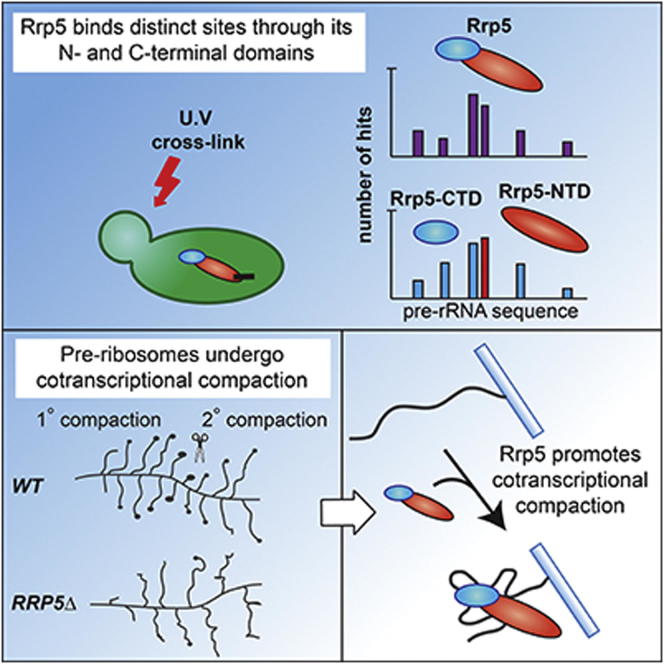

## Introduction

The yeast preribosomal RNA (pre-rRNA) undergoes a complex processing and assembly pathway to generate the mature ribosomal subunits. Three early pre-rRNA cleavages, at sites A0, A1, and A2, generate the 20S pre-rRNA, which is the direct precursor to the 18S rRNA (Figure S1 available online). These cleavages can either occur posttranscriptionally following release of the 35S pre-rRNA (released transcript cleavage; RTC) or cotranscriptionally on the nascent transcript (nascent transcript cleavage; NTC) (Kos and Tollervey, 2010; Osheim et al., 2004) (K. Axt and D.T., unpublished data). NTC occurs when the transcribing polymerase has traveled into the 5′ region of the 25S rDNA, around 1.2–1.5 kb downstream of site A2, but the features involved in establishing the timing of this apparent window for NTC are unclear. The first committed step on the major pathway of 5.8S and 25S rRNA maturation is cleavage at site A3 by the RNA-protein complex ribonuclease (RNase) MRP (Figure S1). Cleavage at A3 predominately occurs posttranscriptionally, and the timing is linked to events at the 3′ end of the 35S pre-rRNA (Allmang and Tollervey, 1998), but the nature of the linkage is also unclear.

**Figure 1:**
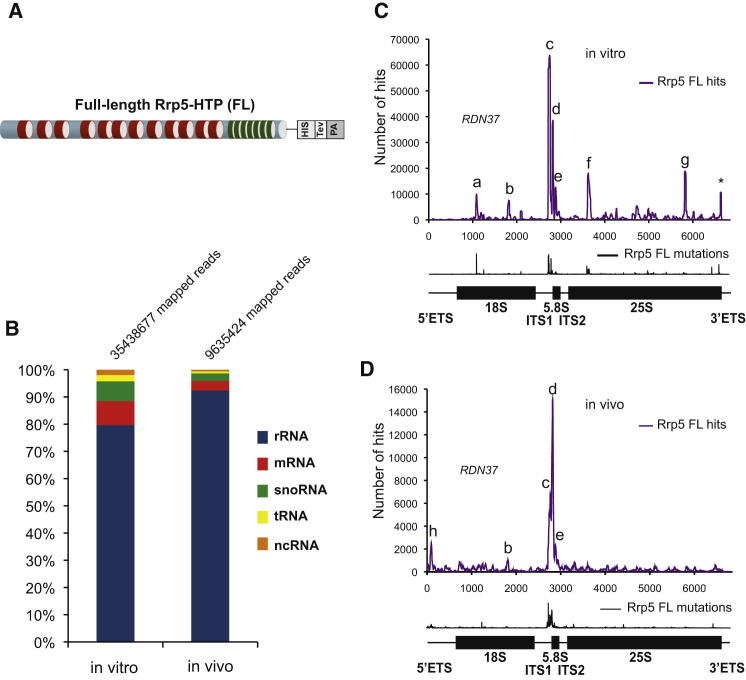
Rrp5 Binding Sites on the Pre-rRNA (A) A translational fusion between Rrp5 and a His6-TEV-ProteinA (HTP) cassette was expressed from the endogenous *RRP5* locus. Rrp5 comprises 12 S1 RNA-binding domains associated with 7 tetratricopeptide repeat (TPR) protein-binding domains. (B) Rrp5-HTP cells were exposed to UV crosslinking while actively growing in culture medium (in vivo) or following cell lysis and clearance of cell debris (in vitro). Crosslinked RNAs were trimmed and ligated to linkers followed by RT-PCR amplification and Illumina sequencing. The sequences obtained were aligned with the yeast genome, and identified target RNAs were sorted into functional categories. The percentage of total mapped reads in the sample is shown for each class. A total of 35 M mapped reads were recovered for the in vitro sample and 9 M for the in vitro sample. (C and D) Sequences obtained from in vitro (C) and in vivo (D) experiments were aligned with the rDNA (RDN37-1) encoding 35S pre-rRNA (nucleotides 1–6,858), and the frequency of recovery (hits per million mapped reads) is plotted for each individual nucleotide (shown in purple). Below the graph, the locations of mutations and deletions are shown in black. These generally represent precise binding sites. In the cartoons, the position of the mature 18S, 5.8S, and 25S rRNAs are indicated by thick lines. Peaks (a–h) are labeled. Peaks that are frequently recovered and present in a control experiment are indicated by a star (^∗^). The two major peaks (c and d) are located in the ITS1.

Among the many protein factors that are required for ribosome synthesis in budding yeast, Rrp5 is unusual in being required for maturation of both subunits. Rrp5 is a large, multidomain protein that contains 12 S1 RNA binding domains and 7 TPR motifs, which are predicted to be protein-protein interaction domains (Figure 1A) (Torchet et al., 1998; Venema and Tollervey, 1996; Young and Karbstein, 2011). Genetic depletion of Rrp5 inhibits the three early cleavages at sites A0, A1, and A2 on the 18S rRNA synthesis pathway and at site A3 on the pathway of 5.8S/25S rRNA synthesis (Venema and Tollervey, 1996). However, loss of Rrp5 does not inhibit the processing of B1L on the alternate pathway of 5.8S/25S processing (see Figure S1), showing the effects on A3 cleavage to be specific. Rrp5 is essential, but its roles in 18S and 5.8S/25S synthesis could be distinguished by coexpression of the separated N-terminal domain (NTD) and C-terminal domain (CTD) regions (Eppens et al., 1999; Torchet et al., 1998; Venema and Tollervey, 1996). The NTD used included S1 RNA-binding domains (RBDs) 1–9, whereas the CTD included S1 RBDs 10–12 and the 7 TPR domains (Eppens et al., 1999). In such strains, depletion of the N-terminal domain blocked cleavage at site A3, whereas depletion of the C-terminal domain inhibited cleavage at sites A0–A2. The domain structure of Rrp5 and its roles in both 40S and 60S subunit synthesis suggested that it might play an important role in coordinating events at multiple sites during early preribosome assembly. However, the actual binding sites and functions of Rrp5 remained unclear.

Rrp5 was initially identified through synthetic-lethal interactions with loss of the snR10 small nucleolar RNA (snoRNA). snR10 directs conversion of uridine to pseudouridine at U2923 in the A loop of the peptidyl transferase center (PTC) region of 25S rRNA (Ganot et al., 1997; Liang et al., 2010; Ni et al., 1997) but is also required for efficient cleavage at sites A0–A2 (Tollervey, 1987). Further genetic and physical interactions were reported between Rrp5 and the RNA helicase Rok1 (Torchet et al., 1998; Young et al., 2013). Mutation of Rok1 is also synthetic lethal with loss of snR10 (Venema et al., 1997), and Rok1 is required for the release of another snoRNA (snR30) from preribosomes (Bohnsack et al., 2008). However, the relationship between the function of Rrp5 and snR10 or snR30 was unclear.

To better understand the functions of Rrp5, we applied UV crosslinking and analysis of cDNA (CRAC) (Granneman et al., 2009) to determine the binding sites of intact Rrp5 and of the separated N-terminal and C-terminal fragments. The major Rrp5 binding sites were identified adjacent to sites A2 and A3. Multiple additional binding sites were identified within the precursors to both subunits and in the snoRNAs that are required for pre-rRNA cleavage.

## Results

### Rrp5 Has Multiple Binding Sites on the Pre-rRNA

A His6-TEV-Protein A (HTP) cassette was inserted at the C-terminal end of the *RRP5* gene in strain BY4741 (Figure 1A). Growth of this strain was similar to BY4741, indicating that the tagged Rrp5-HTP is functional. UV crosslinking was performed under two different conditions. For in vitro crosslinking, the cells were harvested by centrifugation, lysed, and Rrp5-HTP was partially purified prior to crosslinking in a Stratalinker (Granneman et al., 2009). This in vitro approach mimics the conditions used for many previous analyses of the protein and RNA composition of preribosomes. For in vivo crosslinking, Rrp5-HTP and the nontagged control strain were UV irradiated while actively growing in culture medium (Wlotzka et al., 2011). In each protocol, Rrp5-HTP was subsequently purified under very stringent conditions, including washing in 6 M guanidine HCl. Associated RNA was partially degraded, ligated to 5′ and 3′ linkers, amplified by RT-PCR, and identified by Illumina sequencing. Analyses presented in Figure 1B show the distribution of recovered sequences among different RNA classes. In both cases, most crosslinked sequences were derived from the pre-rRNA (80% in vitro and 91% in vivo), with lower numbers of hits on mRNAs (9% and 3%) and snoRNAs (7% and 3%).

The distribution of crosslinked sites along the pre-rRNA is shown in purple in Figures 1C and 1D. For this analysis, all duplicated, identical sequences, which might represent PCR artifacts, have been combined, so the graph shows the distribution of unique sequence reads. The highest hit density was in the internal transcribed spacer 1 (ITS1) region, but additional minor peaks were also observed. The peaks were annotated as sites (a)–(h), with the same letter used to indicate the binding site in different data sets. The significance of these hits was supported by the identification of point mutations (shown in black in lower graphs), which indicate the locations of the direct protein-RNA contacts. Peaks identified by in vitro crosslinking (Figure 1C) were more widely distributed than those found by in vivo crosslinking (Figure 1D). The cells were cooled during harvesting, prior to in vitro crosslinking, and we speculate that slowed cell metabolism led to increased recovery of interactions between Rrp5 and target RNAs that are normally very transient.

### Rrp5 Binding Sites Are Partitioned between the NTD and CTD Regions

**Figure 2:**
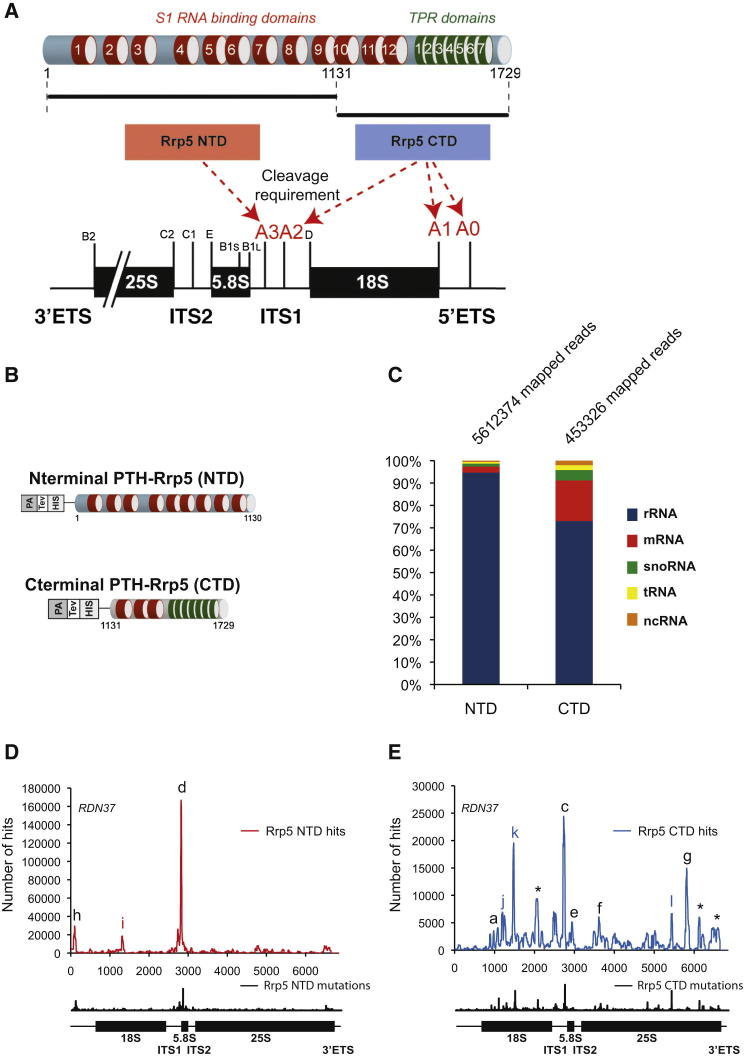
Binding Sites for the Rrp5 NTD and CTD Regions (A) Schematic of the domain structure of Rrp5 and functional targets of the NTD and CTD. Red arrows indicate the requirements for these domains in the pre-rRNA cleavages. Note that for clarity, the pre-rRNA is drawn in a 3′-5′ orientation. (B) Schematic of the tagged versions of the N- and C-terminal domains of Rrp5. (C) NTD: *P*_*GAL*_*::RRP5* with plasmids expressing PTH-Rrp5NTD (Rrp5 aa 1–1,130) and untagged Rrp5CTD. CTD: *P*_*GAL*_*::RRP5* with plasmids expressing PTH-Rrp5CTD (Rrp5 aa 1,131—1,729) and untagged Rrp5NTD. Strains were grown in glucose medium and UV crosslinked in vivo, and RNA sequences were obtained and analyzed as in Figure 1. The percentage of total mapped reads in the sample is shown for each class of RNA. A total of 5.6 M mapped reads were recovered for the NTD and 0.5 M for the CTD. (D and E) rDNA sequences were treated as in Figure 1. Peaks that are frequently recovered and present in a control experiment are indicated by an asterisk (^∗^). Peaks for the N- and C-terminal domains with the same coordinates as found for the full-length Rrp5 are indicated by the same letter in black. A peak of sequences recovered specifically with Rrp5NTD-HTP is labeled i. Peaks of sequences recovered specifically with the Rrp5CTD-HTP are labeled j, k, and l. Mutations are indicated as for Figures 1C and 1D.

The NTD and CTD of Rrp5 are required for different pre-rRNA processing events (Eppens et al., 1999) (Figure 2A). This suggested that they might also have distinct binding sites. To assess this, strains were constructed that expressed the separated N-terminal and C-terminal regions of Rrp5, in which either the NTD or the CTD carried an N-terminal protein A-TEV-His6 (PTH) tag (Figure 2B). Coexpression of the two halves with or without a tag from plasmids supported growth of a *P*_*GAL*_*::RRP5* strain depleted of endogenous Rrp5, but with an increased doubling time (Rrp5, 2 hr; PTH-Rrp5NTD, 2.5 hr; PTH-Rrp5CTD, 3 hr). Strains expressing tagged Rrp5NTD or Rrp5CTD were crosslinked in vivo. For both the NTD and CTD fragments, most hits were recovered in the pre-rRNA (Figure 2C); however, the CTD data set included a greater portion of hits in mRNAs and snoRNAs. The increased recovery of mRNA sequences may reflect increased background, since the CTD is reported to show weaker pre-rRNA association (Young and Karbstein, 2011). However, the increased recovery of snoRNAs appears to largely reflect association with functionally relevant species (see below).

The distributions of NTD and CTD hits along the pre-rRNA are shown in Figures 2D and 2E. As for Rrp5 full-length HTP, the highest density was within ITS1 for both data sets. Notably, binding sites (a)–(h), which were identified in the full-length data sets, were clearly partitioned between the Rrp5NTD and Rrp5CTD targets, with most binding sites associated with the CTD. Four additional sites were detected, designated (i) in the PTH-Rrp5NTD data set or (j), (k), and (l) in the PTH-Rrp5CTD data set. Overall, the combined binding sites recovered with the two fragments of Rrp5 in vivo more closely resembled the in vitro pattern for the full-length protein. The reduced growth supported by the separated domains of Rrp5 suggests that pre-rRNA processing is slower in this strain, and we speculate that this leads to better recovery of normally transient interactions. Northern analysis showed 35S and 23S pre-rRNA accumulation in this strain, indicating a substantial delay in cleavage sites A0–A2 and the accumulation of preribosomal particles (Figure S2).

**Figure 3:**
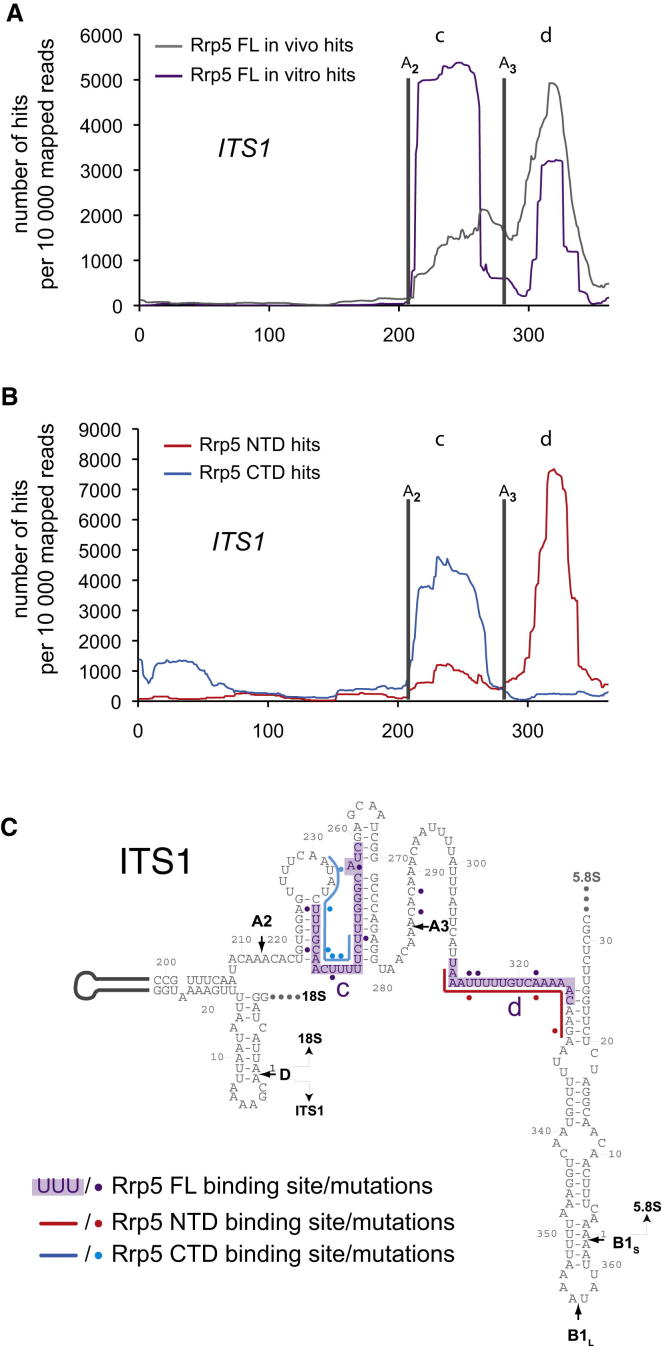
Sites of Rrp5 Association with ITS1 (A) Mapped reads for Rrp5-HTP full-length in vitro (purple) and in vivo (gray). (B) Mapped reads for Rrp5NT-HTP (red) and Rrp5CT-HT (blue) in vivo. The graphs show hit density for each nucleotide over the ITS1 region, per 10,000 total reads mapped within ITS. Processing sites A_2_ and A_3_ are indicated on the graphs, as well as the major peaks annotated as c and d. (C) Predicted secondary structure of the yeast ITS1 region. The binding sites identified in vitro for full-length Rrp5-HTP (purple) and in vivo for Rrp5NTD-HTP (red) or Rrp5CTD-HTP (blue) are indicated. Mutated nucleotides are designated by dots alongside the sequence, using the same colors.

In all experiments, the major hits were found in ITS1, and we analyzed these interactions in more detail (Figures 3 and S3). Full-length Rrp5-HTP showed two prominent peaks of crosslinking, located 3′ to cleavage site A2 and 3′ to cleavage site A3, both in vitro and in vivo (Figure 3A). These binding sites lie within larger regions previously reported to associate with Rrp5 in vivo (de Boer et al., 2006) or in vitro (Young and Karbstein, 2011). To address the contribution of the two domains of Rrp5 to binding at these sites, we analyzed the binding of PTH-Rrp5NTD and PTH-Rrp5CTD to ITS1 (Figure 3B). Strikingly, the NTD, which is required for pre-rRNA cleavage at site A3, was strongly crosslinked 3′ to A3 (Figure 3B). In contrast, the CTD, which is required for pre-rRNA cleavage at site A2, was strongly crosslinked 3′ to A2 (Figure 3B). The significance of these interactions was supported by the identification of point mutations (Figure 3C), which indicate the locations of direct protein-RNA contacts (Granneman et al., 2009; Licatalosi and Darnell, 2010; Wlotzka et al., 2011). Notably, the same major sites for point mutation were generally found for full-length Rrp5 and for the Rrp5NTD or Rrp5CTD binding at the corresponding sites (Figure 3C). This indicates that the NTD and CTD regions of Rrp5 form the same interactions as the intact protein, clearly indicating functional separation.

**Figure 4:**
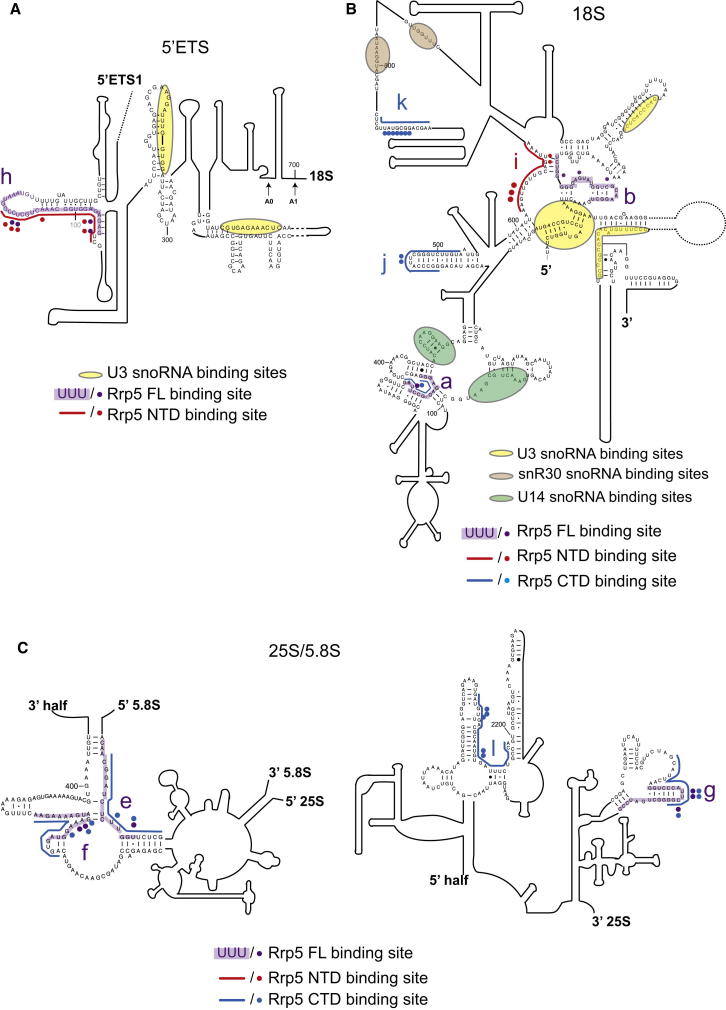
Sites of Rrp5 Association with the Pre-rRNA Predicted secondary structure of part of the 5′ ETS, 18S, 5.8S, and 25S regions. The binding sites of full-length Rrp5-HTP (purple), Rrp5NT-HTP (red), and Rrp5CT-HTP (blue) are indicated on the sequences. Mutated nucleotides found in the sequences of full-length Rrp5-HTP (purple), Rrp5NT-HTP (red), and Rrp5CT-HTP (blue) are indicated by dots alongside the sequence. Binding sites for snoRNAs U3 (yellow), U14 (green), and snR30 (brown) are also indicated.

Outside the A2-A3 region of the pre-rRNA, the major binding sites of Rrp5NTD and Rrp5CTD were also distinct. To assess their potential significance, these additional binding sites and the corresponding point mutations were mapped onto the predicted pre-rRNA secondary structure (Figure 4). Full-length Rrp5 and the Rrp5NTD were bound to the +100 region of the 5′-ETS site (h) (Figure 4A); no specific function has previously been assigned to this region of the pre-rRNA.

Several binding sites were identified within the 18S rRNA (Figure 4B). Site (a) was bound by Rrp5 and Rrp5-CTD and is close to the binding sites for the U14 snoRNA, which is required for processing at sites A0, A1, and A2 (Enright et al., 1996; Lempicki et al., 1990; Li et al., 1990). Site (b) is adjacent to the 3′ side of the central pseudoknot, a long-range interaction that is a key structural feature of the small ribosomal subunit. Crosslinking at site (b) was observed only for full-length Rrp5, but a binding site for Rrp5NTD (i) is in close proximity, as is a binding site for the U3 snoRNA that is also required for A0–A2 processing. Sites (j) and (k) were found only with the Rrp5CTD. Site (k) is located in expansion segment ES6 close to binding sites for snR30, another snoRNA required for cleavage at A0, A1, and A2. The correlation observed between binding sites in 18S rRNA for Rrp5 and for snoRNAs involved in pre-rRNA cleavage at sites A0–A2 strongly indicates that these factors work together during the early steps in ribosome assembly.

Within the 25S and 5.8S rRNAs (Figure 4C), sites (e) and (f) are located at the 5.8S:25S interface and were recovered with full-length Rrp5 and the CTD, suggesting a potential role for Rrp5 in establishing 5.8S:25S rRNA base-pairing. Further 3′ in the 25S rRNA, site (g) around +2,550 was bound by full-length Rrp5 and Rrp5CTD, while site (l) around +2,200 was recovered only with the CTD. We speculate that binding of Rrp5CTD within 25S rRNA may influence the timing of cotranscriptional A0–A2 processing, which takes place only when transcription has extended into the 25S rRNA region (Kos and Tollervey, 2010) (K. Axt and D.T., unpublished data).

### Rrp5 Dissociates from Preribosomes following A3 Cleavage

To assess the timing of the association of Rrp5 and its domains with the pre-rRNA, immunoprecipitation (IP) was performed using Rrp5-HTP, PTH-Rrp5NTD, or PTH-Rrp5CTD. Coprecipitated RNAs were analyzed using the probes indicated (Figure S2A) in primer extension and northern hybridization (Figures S2B and S2C). As controls, IPs were performed with the early pre-40S binding factor Mrd1, the early pre-60S factor Nop7-HTP, and late 60S assembly factor Arx1-HTP (Figures S2B and S2C).

Primer extension analysis (Figure S2B) showed interactions for Rrp5 with the 35S pre-rRNA, with the 33S pre-RNA resulting from A0 cleavage, and with the 32S/20S pre-rRNAs that have 5′ ends at A1 (see processing pathway in Figure S1). Cleavages at sites A2 and A3 generate the 5′ ends of the 27SA2 and 27SA3 pre-rRNAs, respectively, and both species were coprecipitated with full-length Rrp5. In contrast, no coprecipitation was seen for the later 27SB or 7S species with 5′ ends at sites B1L and B1S or for the 26S pre-rRNA with a 5′ end at site C2.

Northern hybridization (Figure S2C) confirmed that Rrp5 is associated with 27SA, but not with the 27SB, 7S, or 20S pre-rRNAs. The failure to recover the 20S species shows that Rrp5 remains associated with the maturing large ribosomal subunit after cleavage at A2. The 27SA and 27SB pre-rRNAs are not clearly resolved by probe 4 in Figure S2C. However, comparison to the Nop7 signal with probe 4 (representing 27SA plus 27SB) and probe 3 (27SA specific) clearly indicates that Rrp5 coprecipitates only 27SA. A3 cleavage is not cotranscriptional, but follows transcript release by cleavage at site B0, 3′ to the 25S rRNA, by the RNase III homolog Rnt1. We conclude that Rrp5 remains associated with the nascent transcript after A2 cleavage but is rapidly released from the pre-60S particles following cleavage at sites B0 and A3.

As expected, the pre-40S factor Mrd1 coprecipitated the early 35S, 33S, and 32S/20S pre-rRNAs, whereas Nop7 coprecipitated the pre-60S species 27SA2, 27SA3, 27SB, 26S, and 7S. Arx1, which acts later in 60S subunit maturation, coprecipitated 27SB, 26S, and 7S. The separated Rrp5NTD and Rrp5CTD fragments coprecipitated the early 35S, 33S, and 32S/20S pre-rRNA, but did not clearly recover 27SA2 or 27SA3 (Figures S2B and S2C). It may be that the separated domains more readily dissociate from the pre-rRNA during the prolonged incubations required for immunoprecipitation.

### Rrp5 Is Associated with Specific snoRNAs

**Figure 5:**
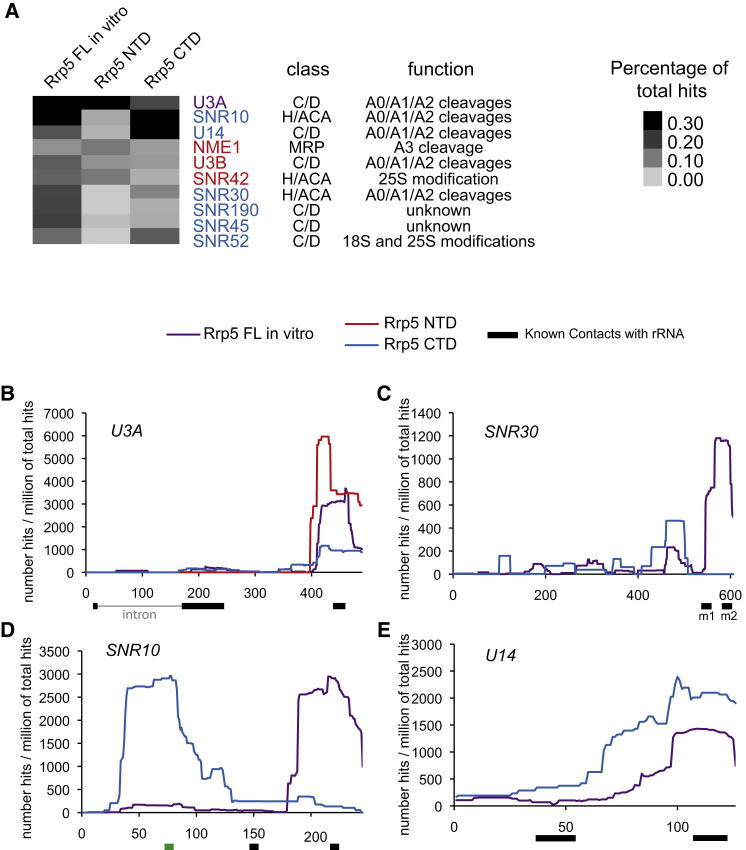
Association of Rrp5 with snoRNAs (A) The snoRNAs identified in each of the data sets were clustered by relative recovery, using Cluster 3.0 and Java TreeView. Recovery as a fraction of total mapped reads is represented in the heatmap. Only snoRNAs that were significantly enriched in more than one sample and in at least one full-length Rrp5-HTP set are included. For full analysis, see Figure S2. Colored names indicate snoRNAs recovered with both Rrp5NTD-HTP and Rrp5CTD-HTP (purple), with only Rrp5NT-HTP (red), and with only Rrp5CT-HTP (blue). Classes of snoRNA (C/D or H/ACA) and functions, where known, are indicated. (B–E) Mapped reads for Rrp5-HTP (purple), Rrp5NTD-HTP (red), and Rrp5CTD-HTP (blue) were aligned with different snoRNAs, and the number of hits for each individual nucleotide is plotted per million mapped reads. Black bars below indicate known regions of interaction with the pre-rRNA. The green bar below SNR10 indicates a 7 nt region required for A0, A1, and A2 cleavage (Liang et al., 2010). In the case of U3A, the region shown is the genomic sequence, which includes a 5′-proximal intron (thin gray line), which splits the 5′ rRNA binding region (thick line).

Rrp5 was crosslinked to the pre-rRNA in close proximity to binding sites for each of the snoRNAs that are required for pre-rRNA cleavage. The snoRNA sequences present in each data set were reported as the percentage of total mapped reads. The distributions of snoRNA hits between the different data sets (Rrp5-HTP in vitro, PTH-Rrp5NTD, and PTH-Rrp5CTD) were used to cluster the snoRNAs. The percentage of total sequences corresponding to different snoRNAs in each data set is represented by heatmaps (Figures 5A and S3A).

Strikingly, three snoRNAs that participate in cleavage of sites A0, A1, and A2 (U3A, U14, and snR10) were grouped together as the most abundant snoRNAs in the data set and were associated with full-length Rrp5 and Rrp5CTD (Li et al., 1990; Hughes and Ares, 1991; Morrissey and Tollervey, 1993). NME1, the RNA component of RNase MRP that cleaves site A3 (Lygerou et al., 1996; Lygerou et al., 1994; Schmitt and Clayton, 1993), was preferentially associated with the Rrp5NTD domain, which is also required for A3 cleavage. NME1 clustered with U3B (which is ∼10-fold less abundant than U3A) and snR42, which directs pseudouridine formation in 25S rRNA (U2975). The significance of the apparent distinction between U3A and U3B is unclear. However, U3 is the only duplicated snoRNA in yeast, and functional differences are possible.

Like snR10 and U14, snR30 is associated with Rrp5 and Rrp5CTD. However, snR30 was recovered less frequently and clustered with the snoRNAs snR190 (cotranscribed with U14 and predicted to bind 18S rRNA, function unknown), snR45 (function unknown), and snR52 (directs methylation of both 18S and 25S rRNA). The remaining snoRNAs are presented in Figure S3A. To understand the interaction of Rrp5 with key snoRNAs required for processing at A0, A1, and A2 (U3, U14, snR10, and snR30) and at A3 (NME1), we analyzed the localization of the Rrp5 binding sites on these snoRNAs (Figures 5B–5E). Rrp5, Rrp5CTD, and Rrp5NTD bind at the 3′ extremity of the U3 snoRNA, far from its major regions of interaction with rRNA (Figure 5B). However, recent analyses of snoRNA interactions by crosslinking and sequencing of hybrids (CLASH) identified interactions between this 3′ region of U3 and two sites within the 18S rRNA (Helwak et al., 2013; Kudla et al., 2011). Both of these U3-18S interactions were recovered multiple times with Rrp5-HTP, providing strong independent confirmation of their formation in vivo (data not shown).

On snR30 (Figure 5C), Rrp5 bound over the m1 and m2 rRNA binding sites, whereas the Rrp5CTD showed some displacement. For snR10 (Figure 5D), the binding sites of Rrp5 and Rrp5CTD appear quite distinct, but notably, the CTD binding region includes a 7 nt sequence required for processing at sites A0, A1, and A2 (Liang et al., 2010), and some binding of full-length Rrp5 was also found in the same region. For U14 (Figure 5E), Rrp5 and Rrp5CTD both associated close to the 3′ interaction site with 18S rRNA.

On NME1, the RNA component of RNase MRP, the major binding sites for Rrp5 and Rrp5NTD were distinct in the primary sequence but are very close in the predicted secondary structure (Figures S3B and S3C). The sites are on opposite sides of the same stem, presumably reflecting subtle differences in protein contacts made by the intact and truncated proteins. The Rrp5NTD target region is essential for NME1 function in yeast (Shadel et al., 2000; Walker and Avis, 2004). The association of the Rrp5NTD with both site A3 in the pre-rRNA and the NME1 RNA is likely to be related to its role in A3 cleavage. To test whether Rrp5 is required to recruit RNase MRP to preribosomal particles, we performed sucrose gradient analyses on cells depleted of Rrp5 (Figure S3D). As a marker of pre-60S particles, we used a probe directed against the 27S pre-rRNAs. However, no clear change in NME1 localization was observed following Rrp5 depletion (Figure S3E). This indicates that Rrp5 is not required for recruitment or release of RNase MRP, although it is essential for its function.

Unexpectedly, interactions were found with the spliceosomal snRNAs U1 and U2, but not with U4, U5, or U6. These were reproducible, but no direct links have been reported between these snRNAs and ribosome assembly, and no involvement of Rrp5 in pre-mRNA splicing has been established, so their significance is currently unclear.

### Protein Interactions with Rrp5

To identify direct protein partners, Rrp5-HTP was purified with or without RNase treatment, and associated proteins were identified by mass spectroscopy. As control, the same analysis was performed using the splicing factor Brr2. To stabilize protein-protein interactions and allow stringent purification under denaturing conditions (8 M urea), complexes were briefly crosslinked with formaldehyde immediately following binding to the immunoglobulin G (IgG) column. Following extensive column washing, bound complexes were released by brief decrosslinking and incubation with SDS. Following this treatment, a smear of material was observed to migrate above Rrp5 (191 kDa) in SDS-PAGE, and this fraction was analyzed by mass spectrometry. Restricting the analysis to this large-sized fraction increased our confidence that bona fide interactions were being identified, particularly for the smaller proteins. We assume that multiple protein-protein crosslinks form in each large preribosomal complex and that these are not fully reversed in the time needed to obtain a useable yield of Rrp5 released from the column. Some degree of reversal of the crosslinks is presumably needed prior to gel analysis, since the intact Rrp5-associated complex is predicted to be >1 MDa and is unlikely to enter the gel.

Purification without RNase treatment resulted in the recovery of a large number of ribosome synthesis factors and ribosomal proteins from both the small and large subunits (Tables 1 and S1). Many of these are expected to be associated with preribosomal particles, rather than with Rrp5 itself. Consistent with this, RNase treatment substantially reduced the complexity of the proteins recovered (Tables 1 and S1). RNA-independent interactions were recovered for the guanosine triphosphatase (GTPase) Bms1, the ATP-binding protein Kre33, the DEAH box helicase Prp43, and DEAD box helicases Dbp9 and Rok1. Consistent with crosslinking to the snoRNAs, interactions were identified with box C/D snoRNP proteins Nop1, Nop56, and Nop58. Numerous ribosomal proteins were also recovered, supporting the participation of Rrp5 in preribosome assembly. However, the most notable class of Rrp5 interacting factors was a group of large proteins, Utp20, Utp10, Mak21/Noc1, Noc2, Rrp12, and Rix1, which are comprised of HEAT or ARM repeats, alpha-helical, structural domains that are predicted to adopt extended conformations (Dez et al., 2007; Dlakić and Tollervey, 2004; Oeffinger et al., 2004). Additionally, Utp21 contains a WD40 domain, which might scaffold protein interactions.

To confirm these interactions by immunoprecipitation, strains that expressed both Rrp5-HTP and GFP-tagged forms of Utp20, Utp10, Utp21, Kre33, Rrp12, and Prp43 were constructed. Nop58 was used as a representative snoRNP protein. We also tested two later pre-60S factors, Nop12 and Urb1, which are not expected to interact with Rrp5. To provide a distinct purification strategy, the GFP-tagged proteins were bound to α-GFP beads under stringent conditions (500 mM NaCl), with and without prior treatment with RNase A + T1. Coprecipitation of Rrp5 was assessed by western blotting with αTAP antibodies after release from the column (Figure S5). No recovery of Rrp5 was seen in the control strain lacking a GFP-tagged protein, and low recovery was seen for Nop12 and Urb1. Good recovery was seen for Utp10, Utp20, Utp21, Nop58, and Kre33, whereas coprecipitation of Rrp5 with Rrp12 and Prp43 was not clearly above the Nop12 and Urb1 controls. In the case of Prp43, previous analyses have shown that its association with preribosomes is very labile (Lebaron et al., 2005), so the crosslinking is likely to be essential for its recovery in the analysis by mass spectrometry. It is currently unclear whether this is also the case for Rrp12.

We conclude that Rrp5 interacts with a set of very large structural proteins that might form a structural framework for compaction of the preribosomal particles by the NTPases.

### Rrp5 Is Required for Preribosome Compaction

**Figure 6:**
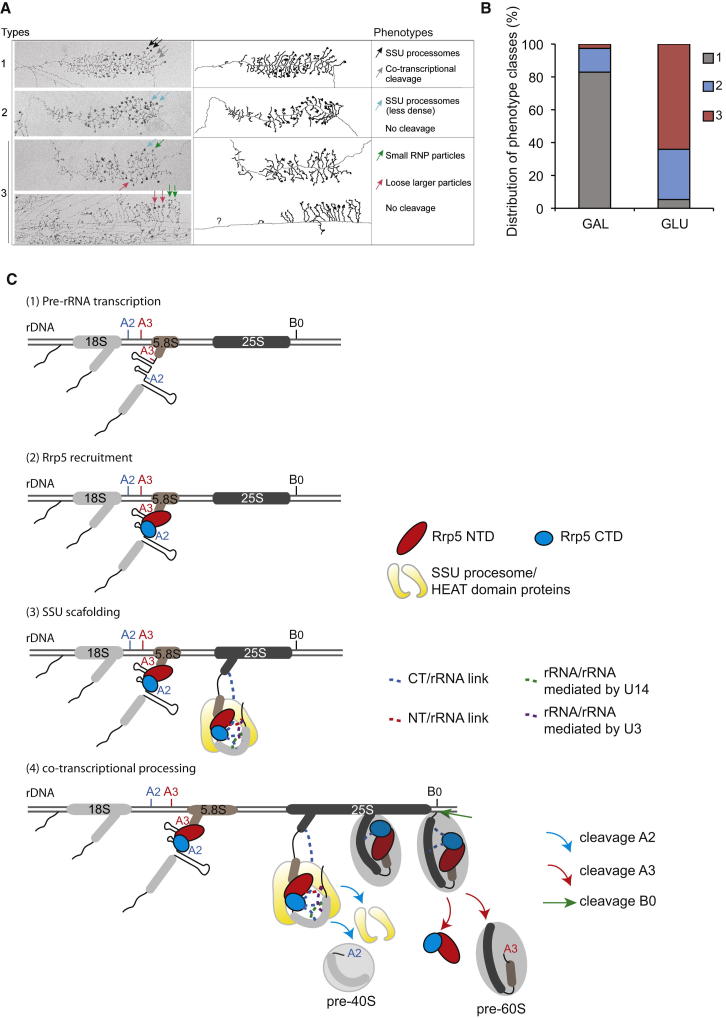
Effects of Rrp5 on Pre-rRNA Compaction and Processing (A) Effects of Rrp5 depletion on pre-rRNA cotranscriptional events were analyzed by EM on chromatin spreads. A *P*_*GAL*_*::RRP5* strain was used, and Miller spreads were prepared from cells growing in galactose medium (n = 228) or following transfer to glucose medium for 4 hr (n = 301). Representative examples of the different phenotypes are presented. Arrows indicate the different cotranscriptional events observed: normal SSU formation (black); normal cotranscriptional cleavage (gray); aberrant, less dense SSU formation (blue); aberrant, small RNP particles (green); aberrant, larger, looser particles (pink). (B) All genes analyzed were assigned to one of three phenotypic categories. Distribution between each category is represented in columns, with normal rDNA morphology indicated in gray. (C) Model for Rrp5 action in pre-rRNA compaction and processing on the major ribosome synthesis pathway. (1 and 2) Rrp5 is a primary preribosome binding factor, and the crosslinking data strongly suggest that the A2 and A3 flanking sequences are the initial binding sites. (3) Rrp5 binds to additional pre-rRNA sites and structural proteins to assemble the large terminal balls that correspond to SSU processome complexes. (4) Following cotranscriptional cleavage at sites A0–A2, Rrp5 remains associated with the nascent transcript until cleavage at site B0. The released pre-rRNA undergoes rapid cleavage at site A3, accompanied or immediately followed by release of Rrp5. For simplicity, the alternative processing pathway of posttranscriptional cleavage at site A2 in the full-length 35S pre-rRNA is omitted.

The many RNA and protein interactions identified for Rrp5 strongly suggested that the 12 S1 RNA binding domains and 7 TPR protein binding domains act to bring together dispersed regions of the pre-rRNA. Compaction of the nascent pre-rRNA into preribosomal particles followed by cotranscriptional cleavage at the A2 cleavage site can be visualized by Miller chromatin spreads of yeast rRNA genes (Gallagher et al., 2004; Miller and Beatty, 1969; Osheim et al., 2004; 2009; Saffer and Miller, 1986). Spreads were prepared from strains expressing Rrp5 under the control of a repressible *P*_*GAL*_ promoter following growth on galactose medium or following transfer to repressive glucose medium for 4 hr (Figure 6A). In analyses of the electron microscopy (EM) images, the individual genes were classified as exhibiting (1) transcripts compacted to form large terminal balls (small subunit [SSU] processomes) accompanied by cotranscriptional cleavage of ∼70% of transcripts on the last quarter of the gene; (2) large terminal balls formed on some transcripts, but generally less dense than on genes in class 1 and with cotranscriptional cleavage largely or entirely absent; and (3), small ribonucleoprotein (RNP) particles visible on some transcripts but morphologically distinct from normal terminal balls and without cotranscriptional cleavage.

The proportion of rDNA repeats in each of these categories was determined on 228 genes from cultures growing in galactose containing medium (expressing Rrp5) and on 301 genes from cultures transferred to glucose media for 4 hr to deplete Rrp5 (Figure 6B). In the presence of Rrp5, >80% of genes fell into class 1. Following depletion of Rrp5, <10% of genes were categorized as class 1. Almost all genes lacked morphologically normal terminal balls, with >60% showing only small RNP particles and most of the remainder showing terminal balls that were less dense than those in wild-type strains, presumably reflecting defective pre-rRNA packaging. Cotranscriptional cleavage was also abolished following Rrp5 depletion. We also tested whether the isolated NTD and CTD domains were able to support preribosome compaction. Examination of EM spreads from *P*_*GAL*_*::RRP5* strains expressing only Rrp5NTD or Rrp5CTD showed that neither domain complemented the Rrp5 depletion phenotype (data not shown).

We conclude that intact Rrp5 is required for pre-rRNA packaging and compaction of the processome into dense terminal balls.

## Discussion

Rrp5 is a highly conserved ribosome synthesis factor that is unusual in being required for the nucleolar maturation of both ribosomal subunits. The presence of multiple RNA binding and protein interaction domains within Rrp5 has long suggested that it might play a key role in the organization and compaction of the preribosomes. From the data presented here, we developed a model for Rrp5 functioning in both pre-rRNA cleavage and guiding cotranscriptional compaction of the nascent pre-rRNA (Figure 6C).

### Distinct Rrp5 Binding Sites with Distinct Functions

The major binding sites for Rrp5 on pre-rRNA were identified in ITS1, adjacent to the A2 and A3 cleavage sites, presumably explaining its dual function in A2 and A3 cleavage. The predominance of these sites in the in vivo data for full-length Rrp5 strongly indicates that these are the primary binding sites, with prolonged binding by Rrp5. We propose that Rrp5 binding at these locations helps define the cleavage sites. Mutational analyses showed that cleavage at site A2 is surprisingly resistant to mutations around the processing site and that definition of the precise position of cleavage is defined in part by 3′ flanking sequences (Allmang et al., 1996). We proposed that this reflected the specific binding of some protein factor, and it now seems very likely that Rrp5 is the relevant factor.

In addition to the major binding sites at A2 and A3, several additional targets were found at locations dispersed over a 6 kb region of the pre-rRNA sequence. When mapped onto the rRNA secondary structure, most of these sites are located in close proximity to functionally important sites for ribosome assembly.

Rrp5 can be physically and functionally separated in N- and C-terminal domains that support Rrp5 function when coexpressed (Eppens et al., 1999; Torchet et al., 1998). To allocate the binding sites recovered for full-length Rrp5 to each domain, we performed CRAC experiments using the separated Rrp5NTD or Rrp5CTD as baits. The separated NTD and CTD regions were not fully functional, supporting a mildly reduced cell doubling time and showing evidence for increased residence time on the pre-rRNA in vivo and reduced stability of pre-rRNA binding in vitro. Despite this, there was a very substantial overlap between the pre-rRNA targets recovered for the intact protein and the separated domains. The NTD is required for cleavage at site A3 and 60S subunit maturation and, strikingly, was strongly bound to the ITS1 region immediately 3′ to site A3. In contrast, the CTD is required for cleavage at A2 and was bound 3′ to this site. These observations offer a potential explanation for the separation of function in the isolated domains.

The cleavages at sites A0–A2 are facilitated by four snoRNAs, U3, U14, snR30, and snR10, which may also participate in pre-rRNA compaction. In particular, the box C/D snoRNA U3 has at least 5 binding sites over the 5′ region of the pre-rRNA. Two of these sites were identified by the CLASH technique for the experimental determination of direct RNA-RNA interaction sites and involved the 3′ stem of U3 base pairing to 18S rRNA sequences in the vicinity of the central pseudoknot (Helwak et al., 2013; Kudla et al., 2011). Rrp5 and the Rrp5CTD were bound to the 3′ stem of U3, and Rrp5 was also crosslinked to the box C/D snoRNP proteins that associate with this region. CLASH analysis of the Rrp5 crosslinking data identified multiple examples of both of these U3-18S rRNA interactions (data not shown). This further confirmed their validity and shows that Rrp5 contacts the U3-18S rRNA complex, rather than free U3.

The box H/ACA class snoRNA snR10 was associated with Rrp5 and the Rrp5CTD domain. Loss of snR10 is synthetic lethal with mutations in Rrp5 and impairs cleavage at sites A0–A2. snR10 targets U2923 in the 25S rRNA for pseudouridine formation, but this site is not yet transcribed when cotranscriptional A2 cleavage occurs. One potential explanation would be that loss of snR10 affects only the subset (∼30%) of transcripts on which cleavage at A2 occurs following transcription termination, but this does not fit well with the published data. It seems more likely that snR10, probably acting in association with Rrp5, has additional binding site(s) in the 5′ region of the pre-rRNA. Notably, a 7 nt sequence of snR10 was shown to be required for cleavage at sites A0, A1, and A2 (Liang et al., 2010), and this region of snR10 was bound by the Rrp5 CTD. However, the short stems formed between the box H/ACA snoRNAs and target RNAs makes them difficult to predict bioinformatically.

The cleavages at sites A0, A1, and A2 are strongly linked genetically and thus might be performed by the same endonuclease. It has been proposed that cleavage of site A2 is mediated by Rcl1 (Horn et al., 2011), but this factor was not identified among the proteins that were chemically crosslinked to Rrp5. A3 cleavage is performed by the RNase MRP particle, and both Rrp5 and the Rrp5NTD were crosslinked to the RNA component of RNase MRP, NME1. Rrp5 is required for A3 cleavage, and the binding site for Rrp5 on NME1 is localized in a region essential for its function. Rrp5 was not required for RNase MRP recruitment to preribosomes, and we propose that Rrp5 activates A3 cleavage by RNase MRP when both are associated with the pre-rRNA.

To better understand the relationship between Rrp5 binding and the cleavage events, we assessed when these interactions occur by immunoprecipitation of full-length Rrp5 or the separated CTD and NTD. These showed that Rrp5 dissociates completely from the 20S pre-rRNA region at A2 cleavage, remaining associated only with the nascent pre-60S RNAs. The 27SA2 pre-rRNA is released from the nascent transcript by Rnt1 cleavage in the 3′ external transcribed spacer region of the pre-rRNA (3′ ETS). The free 27SA2 pre-rRNA is cleaved at site A3 by RNase MRP, and this is rapidly followed by release of Rrp5. Following both A2 and A3 cleavages, Rrp5 must dissociate from multiple binding sites, indicating the presence of an active recycling activity. The timing of A3 cleavage is dependent on events in the 3′ ETS, but the mechanism linking these steps has not been clear (Allmang and Tollervey, 1998). Since Rrp5 is required for A3 cleavage and associates with the 3′ region of the pre-rRNA, it is a plausible candidate to link processes at these distant sites.

### A Structural Framework for Pre-rRNA Folding and Preribosome Assembly?

Analyses of chromatin spreads by EM showed that intact Rrp5 is required for pre-rRNA packaging and compaction of the processome into the terminal balls that normally form the characteristic decorations on Miller “Christmas trees.” Depletion of several other early pre-rRNA processing factors results in the failure to form any sort of large terminal ball. However, the loose processomes seen on Rrp5 depletion were unusual. This probably reflects the observation that many factors required for large processome formation, including the Utp-A/tUtp and Utp-B preribosomal complexes and the U3 snoRNP, can assemble independently of Rrp5 (see Pérez-Fernández et al., 2011). Rrp5 is, however, needed for their compaction into a correctly assembled preribosome.

Protein crosslinking following RNase treatment was used to identify direct protein contacts for Rrp5. Following affinity purification, protein complexes were separated on SDS polyacrylamide gels, and fractions larger than Rrp5 were recovered and analyzed. Due to the large size of Rrp5 (193 kDa), this region has few contaminating proteins, and this, combined with the high stringency of affinity purification and washing of the column under denaturing conditions (8M urea), makes it very likely that most or all of the recovered proteins reflect genuine interactions. Comparison to the similar-sized splicing factor Brr2 confirmed specificity of the Rrp5 interactions. Moreover, the data were supported by western blotting and by recent, independent reports of direct interactions between Rrp5 and Rok1, Noc1, and Noc2 (Hierlmeier et al., 2013; Young et al., 2013).

A striking feature of the proteins that crosslinked to Rrp5 was the identification of a set of large proteins (Utp20, Utp10, Mak21/Noc1, Noc2, and Rix1) that are comprised of HEAT or Armadillo (ARM) repeats. Both of these motifs consist of multiple ∼40 amino acid (aa) alpha-helical bundles that tend to form rigid, highly extended structures. It seems very likely that this set of big, structural proteins acts together with Rrp5 to form the physical core of the large terminal balls that are visualized in EM. In eukaryotes, each pre-rRNA transcript must be bound by many different snoRNAs (75 in yeast), and these interactions predominately occur cotranscriptionally (Kos and Tollervey, 2010). Most snoRNA binding sites are located on sequences that will form the highly folded and compacted core of the ribosomal subunits. This poses a major potential problem, since these sequences must initially be open and exposed for snoRNA binding and then refolded only after modification is complete. Moreover, several different snoRNAs have overlapping binding sites, implying that more than one round of snoRNA binding and release must occur (van Nues et al., 2011). The HEAT and ARM repeat proteins identified would be candidates to form a rigid structural framework that might facilitate unfolding and refolding of the nascent pre-rRNA transcripts, involving the helicases (Prp43, Dbp9, and Rok1) and GTPase (Bsm1) that are also associated with Rrp5. We propose that the large-scale organization for this preribosome refolding is provided by the multiple RNA binding sites of Rrp5. Consistent with this model, binding by Rrp5 is reported to confer target specificity on the Rok1 helicase (Jenner et al., 2012), while Rrp5 binding site (f) overlaps with a binding site for the Prp43 helicase (Bohnsack et al., 2009).

We conclude that the combination of multiple TPR protein-protein interaction domains with numerous S1 RNA binding domains in Rrp5 provides a structural framework to coordinate large-scale RNP folding during ribosome biogenesis.

## Experimental Procedures

### Strains and Oligonucleotides

Strains used in this work are listed in Table S2. Oligonucleotides used are listed in Table S3.

### Crosslinking and Analysis of Illumina Sequence Data

Experiments using full-length Rrp5-HTP were performed on cultures grown to optical density 600 (OD_600_) 0.5 in synthetic dextrose (SD) medium lacking Trp and Ura with 2% glucose. Cells were either directly crosslinked inside culture media using the Megatron (in vivo) (Granneman et al., 2011) or centrifuged, with the pellet suspended in cold PBS before being UV-irradiated using a Stratalinker (in vitro). Both NTD and CTD domains were analyzed by in vivo crosslinking. The number of repeat experiments performed under each condition are indicated in Table S4. Cells were processed as previously described (Granneman et al., 2009; 2010). Illumina sequencing data were aligned to the yeast genome using Novoalign (http://www.novocraft.com). Downstream analyses, including the pileups presented here, were performed using the pyCRAC tool suite (available upon request: sgrannem@staffmail.ed.ac.uk; S.G., unpublished data). Cluster generation used Cluster 3.0 and Java TreeView.

### EM Analysis

For EM analysis of nascent transcript morphology before and after Rrp5 is depleted, strain ASY088, which expresses Rrp5 under the control of a *P*_*GAL*_ promoter, was either grown in yeast extract, peptone, galactose (YPgal) or transferred from YPgal to yeast extract, peptone, dextrose (YPD) for 4 hr prior to chromatin spreading. Miller chromatin spreads were made from cell lysates as described previously (French et al., 2003). Multiple EM grids were examined from three independent experiments for depletion of Rrp5. All rRNA genes visible on these grids were photographed and included in the analysis. Micrographs were visually examined and scored for transcript morphology as described in the legend to Figure 6.

## Figures and Tables

**Table 1:** Summary of Proteins Crosslinked to Rrp5 following RNase Treatment

Gene Name	Protein Name	Structural Domain/Definition	Molecular Weight (kDa)
**40S Synthesis Factors**			

YJL109C	Utp10	HEAT repeats	200.869
YGR090W	Utp22	unknown	141.024
YBL004W	Utp20	HEAT repeats	288.463
YLR409C	Utp21	WD40 domain	105.182
YOR310C	Nop58	core protein of box C/D snoRNP	56.978
YLR197W	Nop56	core protein of box C/D snoRNP	57.057
YDL014W	Nop1	core protein of box C/D snoRNP	34.615
YDL148C	Nop14	unknown	94.415
YNL132W	Kre33	ATP-binding domain	119.615
YPL217C	Bms1	GTPase	135.829
YPL012W	Rrp12	HEAT repeats	138.278
YGL171W	Rok1	DEAD box RNA helicase	64.126
YGL120C	Prp43	DEAH box RNA helicase	87.906

**60S Synthesis Factors**			

YDR060W	Mak21/Noc1	HEAT repeats	116.719
YLR276C	Dbp9	DEAD box RNA helicase	68.302
YKL014C	Urb1	unknown	204.014
YOR206W	Noc2	ARM repeat	81.835
YHR197W	Rix1	ARM repeat	87.126

**Ribosomal Proteins**			

YDR025W	Rps11	40S	17.852
YML024W	Rps17	40S	15.836
YBL072C	Rps8	40S	22.590
YOR096W	Rps7	40S	21.609
YLR048W	Rps0	40S	28.002
YIL069C	Rps24	40S	15.319
YJL190C	Rps22	40S	14.674
YLR167W	Rps31	40S	17.433
YOL121C	Rps19	40S	15.907
YGL135W	Rpl1	60S	24.698
YBL087C	Rpl23	60S	14.578
YGR085C	Rpl11	60S	19.794
YHL033C	Rpl8	60S	28.164
YGL030W	Rpl30	60S	11.408
YPL143W	Rpl33	60S	12.147

**Other Proteins**			

YOR341W	Rpa190	Pol I subunit	187.683
YPR010C	Rpa135	Pol I subunit	136.682
YOR204W	Ded1	DEAD box RNA helicase	65.741
YAL005C	Ssa1	ATPase/chaperone	69.786
YOR133W	Eft1	GTPase/translation	93.686
YPR080W	Tef1	GTPase/translation	50.400

Proteins were included in the list if Rrp5-associated peptides were recovered after RNase treatment in at least two of the three samples, if recovery following RNase treatment was at least 30% of the recovery without RNase, indicating a direct interation, and if recovery was at least 3-fold greater than recovery in Brr2 control samples without RNase. The exception was the box C/D snoRNP proteins (Nop1, Nop56, and Nop58), which are expected to associate with intron-encoded snoRNAs. The association of Rok1, Noc1, and Noc2 with Rrp5 has been reported independently (Hierlmeier et al., 2013; Young et al., 2013).

## Accession Numbers

The GEO accession number for all sequence data reported in this paper is GSE47680.

## Supplemental Information

Document S1. Supplemental Experimental Procedures, Figures S1–S5, and Tables S1–S4

## References

[bib1] Allmang C., Tollervey D. (1998). The role of the 3′ external transcribed spacer in yeast pre-rRNA processing. J. Mol. Biol..

[bib2] Allmang C., Henry Y., Wood H., Morrissey J.P., Petfalski E., Tollervey D. (1996). Recognition of cleavage site A(_2_) in the yeast pre-rRNA. RNA.

[bib3] Bohnsack M.T., Kos M., Tollervey D. (2008). Quantitative analysis of snoRNA association with pre-ribosomes and release of snR30 by Rok1 helicase. EMBO Rep..

[bib4] Bohnsack M.T., Martin R., Granneman S., Ruprecht M., Schleiff E., Tollervey D. (2009). Prp43 bound at different sites on the pre-rRNA performs distinct functions in ribosome synthesis. Mol. Cell.

[bib5] de Boer P., Vos H.R., Faber A.W., Vos J.C., Raué H.A. (2006). Rrp5p, a trans-acting factor in yeast ribosome biogenesis, is an RNA-binding protein with a pronounced preference for U-rich sequences. RNA.

[bib6] Dez C., Dlakić M., Tollervey D. (2007). Roles of the HEAT repeat proteins Utp10 and Utp20 in 40S ribosome maturation. RNA.

[bib7] Dlakić M., Tollervey D. (2004). The Noc proteins involved in ribosome synthesis and export contain divergent HEAT repeats. RNA.

[bib8] Enright C.A., Maxwell E.S., Eliceiri G.L., Sollner-Webb B. (1996). 5’ETS rRNA processing facilitated by four small RNAs: U14, E3, U17, and U3. RNA.

[bib9] Eppens N.A., Rensen S., Granneman S., Raué H.A., Venema J. (1999). The roles of Rrp5p in the synthesis of yeast 18S and 5.8S rRNA can be functionally and physically separated. RNA.

[bib10] French S.L., Osheim Y.N., Cioci F., Nomura M., Beyer A.L. (2003). In exponentially growing Saccharomyces cerevisiae cells, rRNA synthesis is determined by the summed RNA polymerase I loading rate rather than by the number of active genes. Mol. Cell. Biol..

[bib11] Gallagher J.E., Dunbar D.A., Granneman S., Mitchell B.M., Osheim Y., Beyer A.L., Baserga S.J. (2004). RNA polymerase I transcription and pre-rRNA processing are linked by specific SSU processome components. Genes Dev..

[bib12] Ganot P., Bortolin M.L., Kiss T. (1997). Site-specific pseudouridine formation in preribosomal RNA is guided by small nucleolar RNAs. Cell.

[bib13] Granneman S., Kudla G., Petfalski E., Tollervey D. (2009). Identification of protein binding sites on U3 snoRNA and pre-rRNA by UV cross-linking and high-throughput analysis of cDNAs. Proc. Natl. Acad. Sci. USA.

[bib14] Granneman S., Petfalski E., Swiatkowska A., Tollervey D. (2010). Cracking pre-40S ribosomal subunit structure by systematic analyses of RNA-protein cross-linking. EMBO J..

[bib15] Granneman S., Petfalski E., Tollervey D. (2011). A cluster of ribosome synthesis factors regulate pre-rRNA folding and 5.8S rRNA maturation by the Rat1 exonuclease. EMBO J..

[bib16] Helwak A., Kudla G., Dudnakova T., Tollervey D. (2013). Mapping the human miRNA interactome by CLASH reveals frequent noncanonical binding. Cell.

[bib17] Hierlmeier T., Merl J., Sauert M., Perez-Fernandez J., Schultz P., Bruckmann A., Hamperl S., Ohmayer U., Rachel R., Jacob A. (2013). Rrp5p, Noc1p and Noc2p form a protein module which is part of early large ribosomal subunit precursors in S. cerevisiae. Nucleic Acids Res..

[bib18] Horn D.M., Mason S.L., Karbstein K. (2011). Rcl1 protein, a novel nuclease for 18 S ribosomal RNA production. J. Biol. Chem..

[bib19] Hughes J.M.X., Ares M.J. (1991). Depletion of U3 small nucleolar RNA inhibits cleavage in the 5′ external transcribed spacer of yeast pre-ribosomal RNA and impairs formation of 18S ribosomal RNA. EMBO J..

[bib20] Jenner L., Melnikov S., Garreau de Loubresse N., Ben-Shem A., Iskakova M., Urzhumtsev A., Meskauskas A., Dinman J., Yusupova G., Yusupov M. (2012). Crystal structure of the 80S yeast ribosome. Curr. Opin. Struct. Biol..

[bib21] Kos M., Tollervey D. (2010). Yeast pre-rRNA processing and modification occur cotranscriptionally. Mol. Cell.

[bib22] Kudla G., Granneman S., Hahn D., Beggs J.D., Tollervey D. (2011). Cross-linking, ligation, and sequencing of hybrids reveals RNA-RNA interactions in yeast. Proc. Natl. Acad. Sci. USA.

[bib23] Lebaron S., Froment C., Fromont-Racine M., Rain J.C., Monsarrat B., Caizergues-Ferrer M., Henry Y. (2005). The splicing ATPase prp43p is a component of multiple preribosomal particles. Mol. Cell. Biol..

[bib24] Lempicki R.A., Jarmolowski A., Huang G.Y., Li H.V., Fournier M.J. (1990). Mutations in conserved domains of U14 RNA impair 18S ribosomal RNA production in Saccharomyces cerevisiae. Mol. Biol. Rep..

[bib25] Li H.D., Zagorski J., Fournier M.J. (1990). Depletion of U14 small nuclear RNA (snR128) disrupts production of 18S rRNA in Saccharomyces cerevisiae. Mol. Cell. Biol..

[bib26] Liang X.H., Liu Q., Liu Q., King T.H., Fournier M.J. (2010). Strong dependence between functional domains in a dual-function snoRNA infers coupling of rRNA processing and modification events. Nucleic Acids Res..

[bib27] Licatalosi D.D., Darnell R.B. (2010). RNA processing and its regulation: global insights into biological networks. Nat. Rev. Genet..

[bib28] Lygerou Z., Mitchell P., Petfalski E., Séraphin B., Tollervey D. (1994). The *POP1* gene encodes a protein component common to the RNase MRP and RNase P ribonucleoproteins. Genes Dev..

[bib29] Lygerou Z., Allmang C., Tollervey D., Séraphin B. (1996). Accurate processing of a eukaryotic precursor ribosomal RNA by ribonuclease MRP in vitro. Science.

[bib30] Miller O.L., Beatty B.R. (1969). Visualization of nucleolar genes. Science.

[bib31] Morrissey J.P., Tollervey D. (1993). Yeast snR30 is a small nucleolar RNA required for 18S rRNA synthesis. Mol. Cell. Biol..

[bib32] Ni J., Tien A.L., Fournier M.J. (1997). Small nucleolar RNAs direct site-specific synthesis of pseudouridine in ribosomal RNA. Cell.

[bib33] Oeffinger M., Dlakic M., Tollervey D. (2004). A pre-ribosome-associated HEAT-repeat protein is required for export of both ribosomal subunits. Genes Dev..

[bib34] Osheim Y.N., French S.L., Keck K.M., Champion E.A., Spasov K., Dragon F., Baserga S.J., Beyer A.L. (2004). Pre-18S ribosomal RNA is structurally compacted into the SSU processome prior to being cleaved from nascent transcripts in Saccharomyces cerevisiae. Mol. Cell.

[bib35] Osheim Y.N., French S.L., Sikes M.L., Beyer A.L. (2009). Electron microscope visualization of RNA transcription and processing in Saccharomyces cerevisiae by Miller chromatin spreading. Methods Mol. Biol..

[bib36] Pérez-Fernández J., Martín-Marcos P., Dosil M. (2011). Elucidation of the assembly events required for the recruitment of Utp20, Imp4 and Bms1 onto nascent pre-ribosomes. Nucleic Acids Res..

[bib37] Saffer L.D., Miller O.L. (1986). Electron microscopic study of *Saccharomyces cerevisiae* rDNA chromatin replication. Mol. Cell. Biol..

[bib38] Schmitt M.E., Clayton D.A. (1993). Nuclear RNase MRP is required for correct processing of pre-5.8S rRNA in *Saccharomyces cerevisiae*. Mol. Cell. Biol..

[bib39] Shadel G.S., Buckenmeyer G.A., Clayton D.A., Schmitt M.E. (2000). Mutational analysis of the RNA component of Saccharomyces cerevisiae RNase MRP reveals distinct nuclear phenotypes. Gene.

[bib40] Tollervey D. (1987). A yeast small nuclear RNA is required for normal processing of pre-ribosomal RNA. EMBO J..

[bib41] Torchet C., Jacq C., Hermann-Le Denmat S. (1998). Two mutant forms of the S1/TPR-containing protein Rrp5p affect the 18S rRNA synthesis in Saccharomyces cerevisiae. RNA.

[bib42] van Nues R.W., Granneman S., Kudla G., Sloan K.E., Chicken M., Tollervey D., Watkins N.J. (2011). Box C/D snoRNP catalysed methylation is aided by additional pre-rRNA base-pairing. EMBO J..

[bib43] Venema J., Tollervey D. (1996). *RRP5* is required for formation of both 18S and 5.8S rRNA in yeast. EMBO J..

[bib44] Venema J., Bousquet-Antonelli C., Gelugne J.-P., Caizergues-Ferrer M., Tollervey D. (1997). Rok1p is a putative RNA helicase required for rRNA processing. Mol. Cell. Biol..

[bib45] Walker S.C., Avis J.M. (2004). A conserved element in the yeast RNase MRP RNA subunit can participate in a long-range base-pairing interaction. J. Mol. Biol..

[bib46] Wlotzka W., Kudla G., Granneman S., Tollervey D. (2011). The nuclear RNA polymerase II surveillance system targets polymerase III transcripts. EMBO J..

[bib47] Young C.L., Karbstein K. (2011). The roles of S1 RNA-binding domains in Rrp5’s interactions with pre-rRNA. RNA.

[bib48] Young C.L., Khoshnevis S., Karbstein K. (2013). Cofactor-dependent specificity of a DEAD-box protein. Proc. Natl. Acad. Sci. USA.

