# Supplementary material for: Rrp5 Binding at Multiple Sites Coordinates Pre-rRNA Processing and Assembly
Source: Mol Cell. 2013 Dec 12;52(5):707–19. doi: 10.1016/j.molcel.2013.10.017 (PMC3991325; doi:10.1016/j.molcel.2013.10.017)
Supplement: Document S1. Supplemental Experimental Procedures, Figures S1–S5, and Tables S1–S4 [file mmc1.pdf]

Molecular Cell, Volume *52*

Supplemental Information

Rrp5 Binding at Multiple Sites Coordinates Pre-rRNA Processing and Assembly

Simon Lebaron, Åsa Segerstolpe, Sarah L. French, Tatiana Dudnakova, Flavia de lima Alves, Sander Granneman, Juri Rappsilber, Ann L. Beyer, Lars Wieslander, and David Tollervey

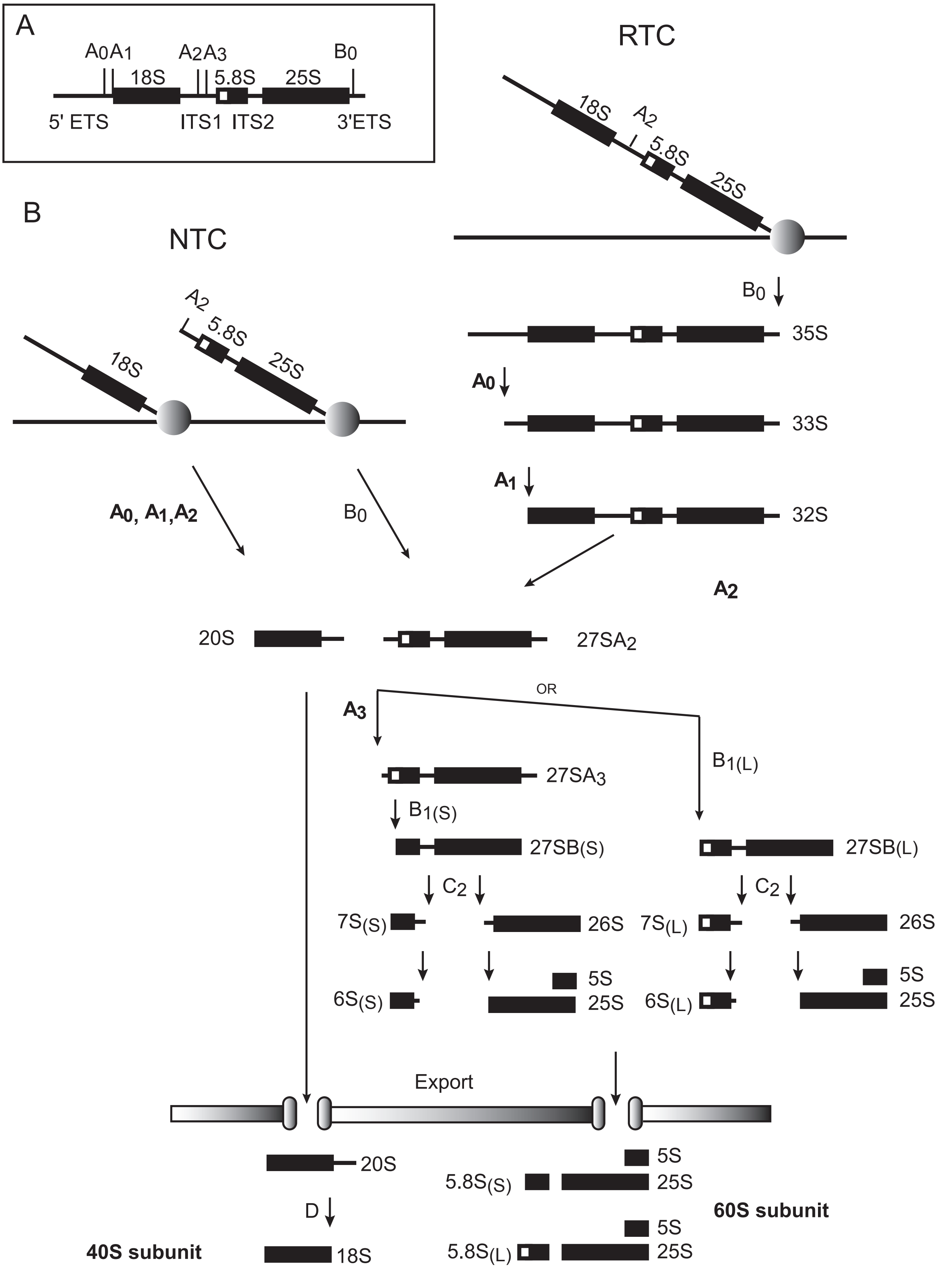
A
A0 A1
A2 A3
B0
18S
5.8S
25S
5' ETS
ITS1
ITS2
3'ETS
B
NTC
RTC
A2
5.8S
18S
25S
B0
35S
A0
33S
A1
32S
A0, A1,A2
B0
A2
20S
27SA2
OR
A3
27SA3
B1(L)
B1(S)
27SB(S)
27SB(L)
C2
C2
7S(S)
26S
7S(L)
26S
5S
5S
6S(S)
25S
6S(L)
25S
Export
20S
5S
5.8S(S)
25S
D
60S subunit
5S
40S subunit
18S
5.8S(L)
25S

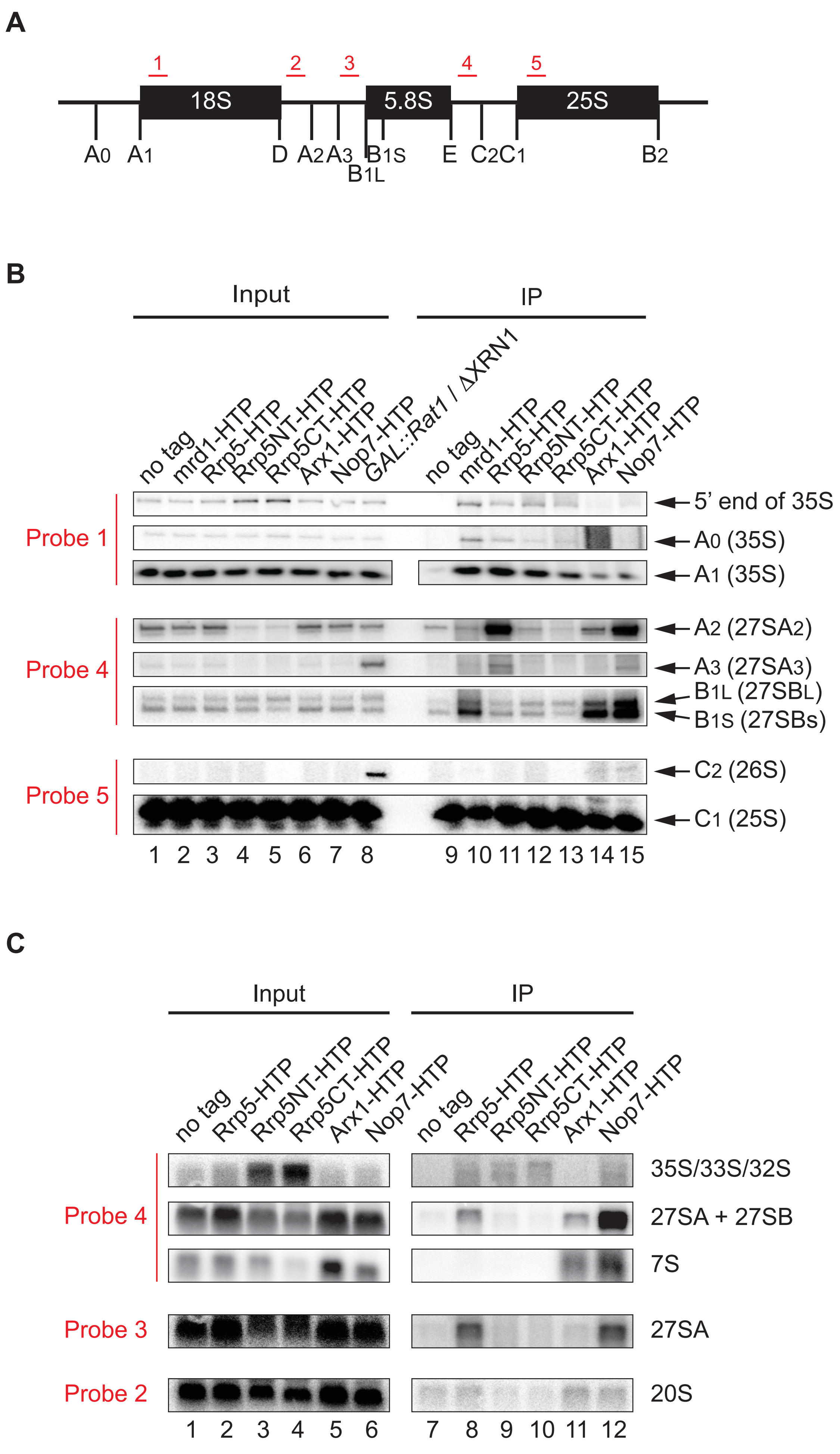
A
1
2
3
4
5
18S
5.8S
25S
A0 A1 D A2 A3 B1S B1L E C2 C1 B2
B
Input
IP
no tag
mrd1-HTP
Rrp5-HTP
Rrp5NT-HTP
Rrp5CT-HTP
Arx1-HTP
Nop7-HTP
GAL::Rat1 / ΔXRN1
Probe 1
Probe 4
Probe 5
5' end of 35S
A0 (35S)
A1 (35S)
A2 (27SA2)
A3 (27SA3)
B1L (27SBL)
B1S (27SBs)
C2 (26S)
C1 (25S)
1 2 3 4 5 6 7 8 9 10 11 12 13 14 15
C
Input
IP
no tag
Rrp5-HTP
Rrp5NT-HTP
Rrp5CT-HTP
Arx1-HTP
Nop7-HTP
Probe 4
Probe 3
Probe 2
35S/33S/32S
27SA + 27SB
7S
27SA
20S
1 2 3 4 5 6 7 8 9 10 11 12

A

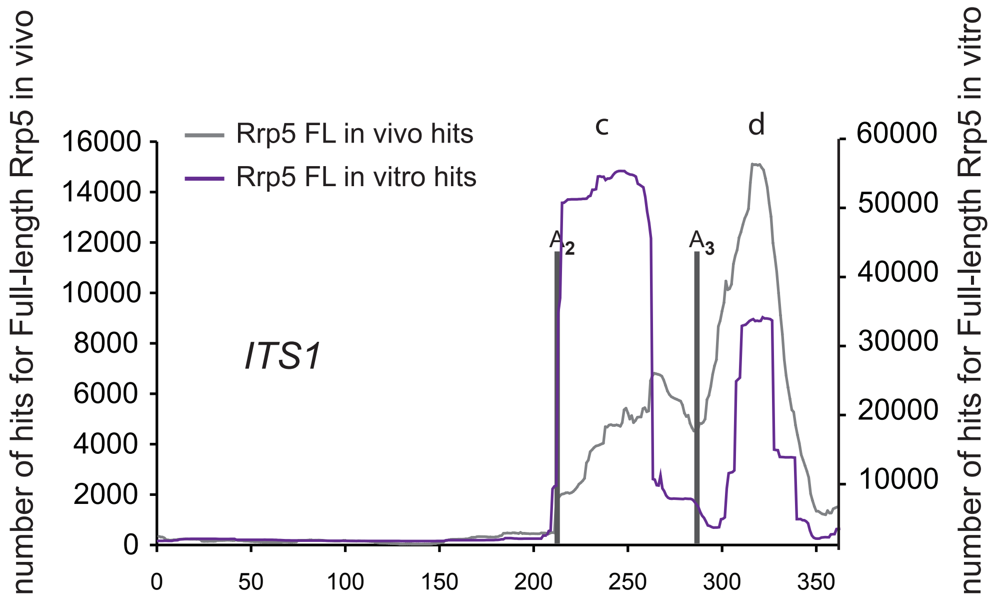
number of hits for Full-length Rrp5 in vivo
16000
14000
12000
10000
8000
6000
4000
2000
0
Rrp5 FL in vivo hits
Rrp5 FL in vitro hits
c
d
A2
A3
ITS1
60000
50000
40000
30000
20000
10000
number of hits for Full-length Rrp5 in vitro
0
50
100
150
200
250
300
350

B

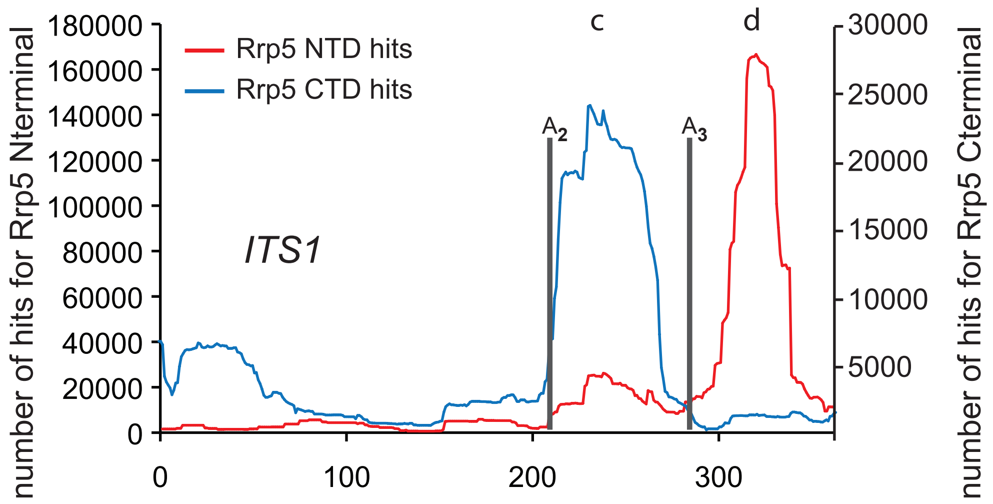
number of hits for Rrp5 Nterminal
180000
160000
140000
120000
100000
80000
60000
40000
20000
0
Rrp5 NTD hits
Rrp5 CTD hits
c
d
A2
A3
ITS1
30000
25000
20000
15000
10000
5000
number of hits for Rrp5 Cterminal
0
100
200
300

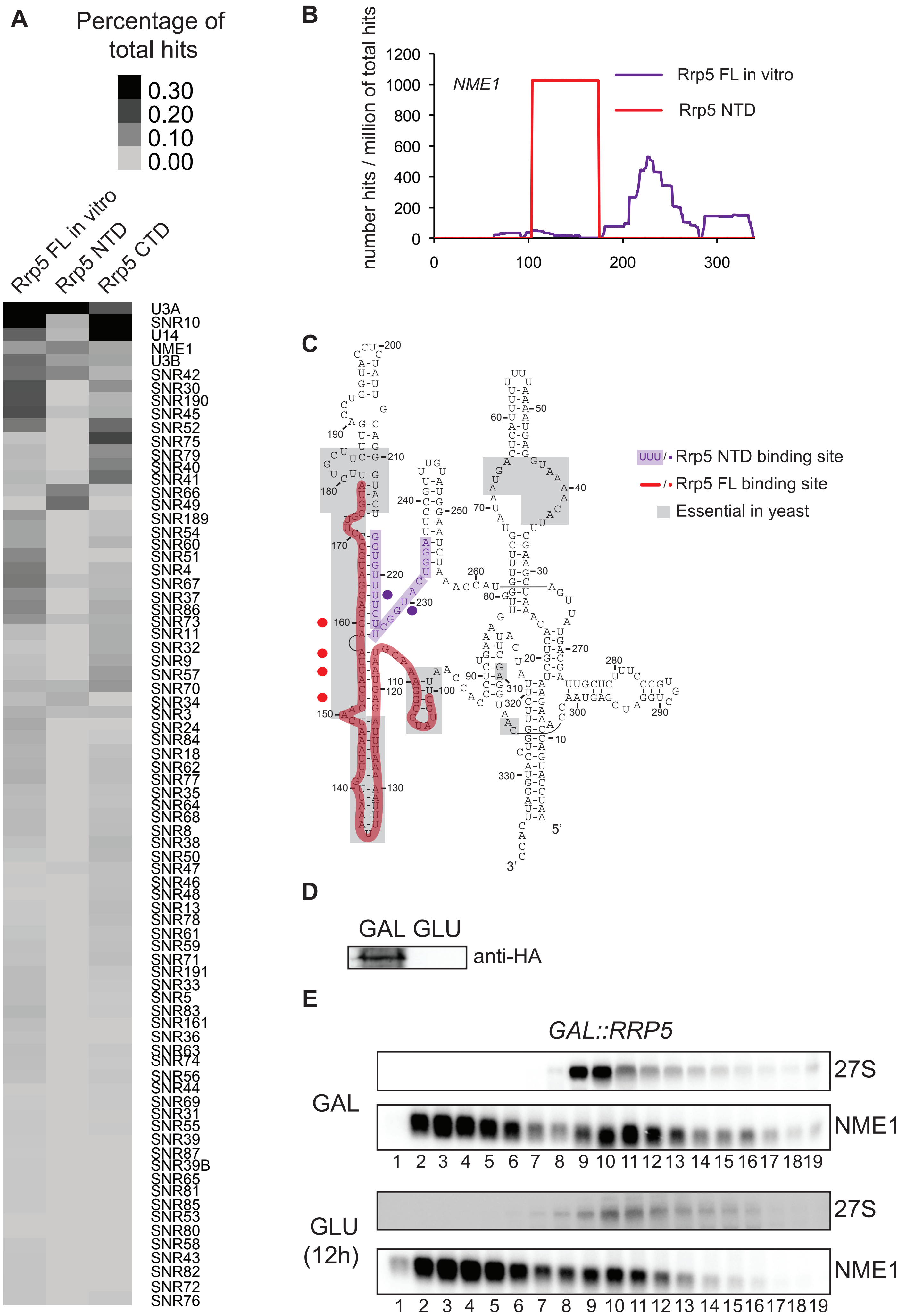
A
Percentage of total hits
0.30
0.20
0.10
0.00
Rrp5 FL in vitro
Rrp5 NTD
Rrp5 CTD
U3A
SNR10
U14
NME1
U3B
SNR42
SNR30
SNR190
SNR45
SNR52
SNR75
SNR79
SNR40
SNR41
SNR66
SNR49
SNR189
SNR54
SNR60
SNR51
SNR4
SNR67
SNR37
SNR86
SNR73
SNR11
SNR32
SNR9
SNR57
SNR70
SNR34
SNR3
SNR24
SNR84
SNR18
SNR62
SNR77
SNR35
SNR64
SNR68
SNR8
SNR38
SNR50
SNR47
SNR46
SNR48
SNR13
SNR78
SNR61
SNR59
SNR71
SNR191
SNR33
SNR5
SNR83
SNR161
SNR36
SNR63
SNR74
SNR56
SNR44
SNR69
SNR31
SNR55
SNR39
SNR87
SNR39B
SNR65
SNR81
SNR85
SNR53
SNR80
SNR58
SNR43
SNR82
SNR72
SNR76
B
number hits / million of total hits
NME1
Rrp5 FL in vitro
Rrp5 NTD
0
200
400
600
800
1000
1200
0
100
200
300
C
Rrp5 NTD binding site
Rrp5 FL binding site
Essential in yeast
5'
3'
D
GAL GLU
anti-HA
E
GAL::RRP5
GAL
GLU
(12h)
27S
NME1
1 2 3 4 5 6 7 8 9 10 11 12 13 14 15 16 17 18 19

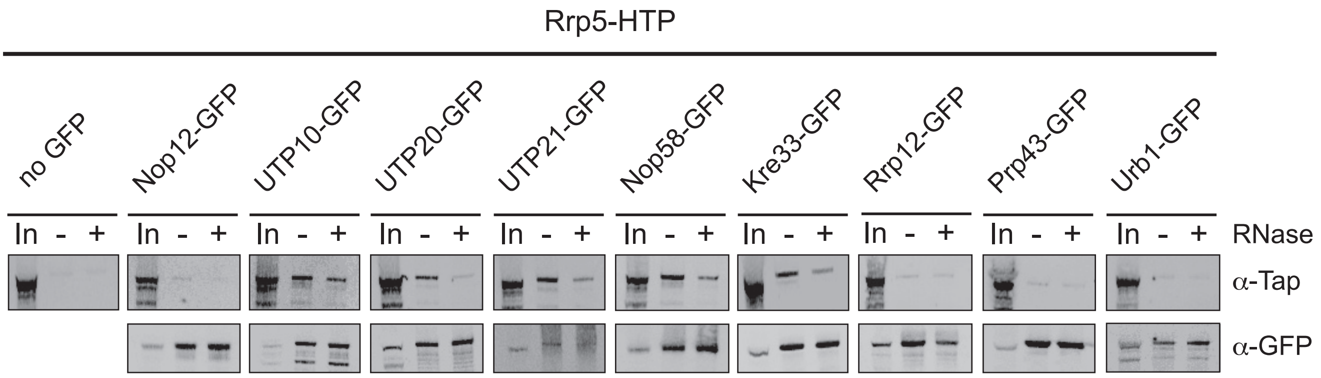
Rrp5-HTP
no GFP
Nop12-GFP
UTP10-GFP
UTP20-GFP
UTP21-GFP
Nop58-GFP
Kre33-GFP
Rrp12-GFP
Prp43-GFP
Urb1-GFP
In - + In - + In - + In - + In - + In - + In - + In - + In - + In - +
RNase
α-Tap
α-GFP

SUPPLEMENTARY FIGURE LEGENDS

Supplementary Figure S1 – related to Figs. 3, 4 and 6 but helpful to readers throughout MS.

Yeast pre-rRNA processing

A: Schematic representation of the yeast pre-rRNA, showing the locations of processing sites most relevant to the results presented. Not to scale.

B: Pre-rRNA processing, showing the alternative pathways of nascent transcript cleavage (NTC) and released transcript cleavage (RTC). In wild-type cells ~70% of pre-rRNA transcripts undergo NTC. Cleavages that are Rrp5-dependent (A0, A1, A2, A3) are indicated in bold.

Supplementary Figure S2 – related to Figs. 1 and 2

Immunoprecipitation of RNAs associated with Rrp5

A: Schematic of the 35S pre-rRNA, with the locations of probes indicated (red).

B: Full-length Rrp5-HTP, Rrp5CT-HTP, Rrp5NT-HTP proteins were used for IP. As controls, a non-tagged strain and tagged forms of proteins involved in early pre-40S processing (Mrd1) and pre-60S processing (Arx1-HTP and Nop7-HTP) were also analyzed. IP was performed and associated RNAs were analyzed by primer extension. Probes are indicated in red. Locations of the primer extension stops and the corresponding pre-rRNA species are indicated in black. The P_{GAL}*::RAT1/xrn1Δ* strain was used in the input as a marker for locations of the C2 and A3 cleavages, which are not readily detected in wild-type pre-rRNA.

C: Following IP the same RNAs were analyzed by Northern-hybridization.

Supplementary Figure S3 - related to Fig 3

Rrp5 hits mapping in ITS1

A: Mapped reads for Rrp5-HTP full length in vitro (purple) and in vivo (grey) were aligned to ITS1. **B:** Reads mapped in ITS1 for Rrp5NT-HTP (red) and Rrp5CT-HT (blue) in vivo. The graphs show hit density for each nucleotide per million total mapped reads. Processing sites A_2 and A_3 are indicated on the graphs as well as the major peaks annotated as c and d.

Supplementary Figure S4 - related to Fig. 5

Analysis of snoRNA hits

A: Full list of snoRNAs recovered.

B: Sequences recovered in the NME1 RNA for the full-length Rrp5-HTP (purple) and

Rrp5NT-HTP (red), expressed as total numbers of hits for each nucleotide per million mapped reads.

C: Predicted secondary structure of NME1 RNA, part essential for its function in A3 cleavage in yeast are indicated in grey. Binding region with full-length Rrp5-HTP (purple) and Rrp5NT-HTP (red) are indicated. Mutated nucleotides found in the sequences of Rrp5-HTP (purple) and Rrp5NT-HTP (red) are indicated by dots.

D: Western-blot showing the depletion of Rrp5 protein after 7h in glucose medium.

E: Analysis of the association of RNase MRP with pre-ribosomes. Extracts from cells expressing Rrp5 (GAL) or following Rrp5 depletion for 7 h (GLU) were fractionated by sucrose gradient centrifugation. Migration of pre-60S ribosomes and RNase MRP was followed by northern hybridization with probes for 27SA pre-RNA and MRP RNA component NME1, respectively .

Supplementary Figure S5 – related to Table 1 and S1

Immunoprecipitation of Rrp5-associated proteins

The association of Rrp5 with protein partners was tested by immunoprecipitation. 10% of the total input extract (In) and proteins recovered following affinity purification on “GFP-trap” beads in the absence (-) or presence of RNase A + T1 (+) were loaded an SDS gel. Western blotting was used to assess Rrp5 binding (anti-TAP) and to verify recovery of the GFP tagged bait protein (a-GFP).

Supplementary Table S1. Summary of mass spectrometry analysis of Rrp5 cross-linking experiment.

Gene Name	Protein Name	Brr2 control 1	Brr2 control 2	Rrp5 No RNase 1	Rrp5 No RNase 2	Rrp5 No RNase 3	Rrp5 No RNase % total peptides	Rrp5 RNase 1	Rrp5 RNase 2	Rrp5 RNase 3	Rrp5 RNase % total peptides	Selection
**40S synthesis factors**												
**YMR229C**	**Rrp5**	**0**	**0**	**139**	**169**	**152**	**26**	**317**	**301**	**222**	**54**	**1**
YER172C	Brr2	76	46	0	0	0	0	17	6	7	1.9	0
**YJL109C**	**Utp10**	**0**	**0**	**9**	**13**	**26**	**2.7**	**9**	**15**	**2**	**1.7**	**1**
**YGR090W**	**Utp22**	**0**	**0**	**1**	**1**	**18**	**1.1**	**38**	**9**	**8**	**3,5**	**1**
**YBL004W**	**Utp20**	**0**	**0**	**0**	**1**	**14**	**0.9**	**3**	**35**	**1**	**2.5**	**1**
**YLR409C**	**Utp21**	**0**	**0**	**3**	**3**	**3**	**0.5**	**1**	**2**	**0**	**0.2**	**1**
YGR128C	Utp8	0	0	1	3	3	0.4	0	1	0	0.1	0
YLR222C	Utp13	0	0	1	3	3	0.4	0	2	0	0.1	0
YMR093W	Utp15	0	0	0	4	3	0.4	0	0	0	0	0
YJL069C	Utp18	0	0	2	1	1	0.2	0	1	0	0.1	0
**YOR310C**	**Nop58**	**3**	**3**	**8**	**11**	**18**	**2.1**	**5**	**17**	**5**	**1.7**	**1**
**YLR197W**	**Nop56**	**2**	**1**	**4**	**14**	**17**	**2.0**	**4**	**8**	**3**	**1.0**	**1**
YPL126W	Nan1	0	0	4	11	12	1.5	0	0	0	0	0
**YDL014W**	**Nop1**	**3**	**2**	**0**	**7**	**10**	**1.0**	**2**	**3**	**1**	**0.4**	**1**
YLR175W	Cbf5	0	5	5	4	6	0.9	2	0	1	0.2	0
YOL041C	Nop12	0	0	8	7	8	1.3	0	0	0	0	0
YLR129W	Dip2	0	0	2	4	7	0.7	0	2	0	0.1	0
**YDL148C**	**Nop14**	**0**	**0**	**2**	**3**	**6**	**0.6**	**1**	**3**	**0**	**0.3**	**1**
YNL061W	Nop2	0	0	0	0	5	0.3	0	0	1	0.1	0
**YNL132W**	**Kre33**	**0**	**0**	**3**	**2**	**8**	**0.7**	**6**	**7**	**2**	**1.0**	**1**
YGR159C	Nsr1	1	0	2	3	14	1.1	0	4	0	0.3	0
**YPL217C**	**Bms1**	**0**	**0**	**2**	**4**	**9**	**0.8**	**2**	**5**	**0**	**0.4**	**1**
**YPL012W**	**Rrp12**	**0**	**0**	**0**	**4**	**14**	**1.0**	**3**	**5**	**0**	**0.5**	**1**
YMR116C	Asc1	0	0	0	0	10	0.6	3	0	0	0,.2	0
**YGL171W**	**Rok1**	**0**	**0**	**1**	**1**	**0**	**0.1**	**9**	**4**	**0**	**0.8**	**1**
**YGL120C**	**Prp43**	**0**	**0**	**0**	**0**	**3**	**0.2**	**3**	**2**	**1**	**0.4**	**1**
YDL007W	Rpt2	0	0	0	0	2	0.1	1	0	0	0.1	0
YKR092C	Srp40	0	0	0	0	1	0.1	0	1	0	0.1	0
**60S Synthesis factors**												
**YDR060W**	**Mak21**	**0**	**0**	**6**	**4**	**12**	**1.3**	**4**	**2**	**4**	**0.6**	**1**

Gene Name	Protein Name	Brr2 control 1	Brr2 control 2	Rrp5 No RNase 1	Rrp5 No RNase 2	Rrp5 No RNase 3	Rrp5 No RNase % total peptides	Rrp5 RNase 1	Rrp5 RNase 2	Rrp5 RNase 3	Rrp5 RNase % total peptides	Selection
YMR049C	Erb1	0	1	5	10	14	1.7	4	0	0	0.3	0
**YLR276C**	**Dbp9**	**0**	**0**	**0**	**1**	**5**	**0.3**	**2**	**17**	**1**	**1.3**	**1**
**YKL014C**	**Urb1**	**0**	**0**	**0**	**0**	**7**	**0.4**	**2**	**3**	**0**	**0.3**	**1**
**YOR206W**	**Noc2**	**0**	**0**	**1**	**1**	**7**	**0.5**	**1**	**0**	**2**	**0.2**	**1**
YLL008W	Drs1	0	0	0	0	9	0.5	0	0	0	0	0
YMR290C	Has1	0	0	0	3	7	0.6	1	1	0	0.1	0
**YHR197W**	**Rix1**	**0**	**0**	**1**	**0**	**0**	**0.1**	**2**	**1**	**0**	**0.2**	**1**
**Ribosomal Proteins**												
**40S**												
YML026C	Rps18	0	1	2	1	4	0.4	2	0	0	0.1	0
**YDR025W**	**Rps11**	**0**	**0**	**2**	**3**	**2**	**0.4**	**4**	**2**	**0**	**0.4**	**1**
**YML024W**	**Rps17**	**0**	**0**	**1**	**2**	**5**	**0.5**	**1**	**2**	**2**	**0.4**	**1**
**YBL072C**	**Rps8**	**0**	**1**	**1**	**4**	**2**	**0.4**	**0**	**4**	**2**	**0.4**	**1**
**YOR096W**	**Rps7**	**0**	**0**	**1**	**0**	**1**	**0.1**	**8**	**3**	**0**	**0.7**	**1**
YPL081W	Rps9	1	1	2	1	5	0.5	0	1	0	0.1	0
**YLR048W**	**Rps0**	**0**	**0**	**0**	**0**	**3**	**0.2**	**7**	**0**	**1**	**0.5**	**1**
**YIL069C**	**Rps24**	**0**	**0**	**0**	**0**	**2**	**0.1**	**5**	**1**	**2**	**0.5**	**1**
**YJL190C**	**Rps22**	**0**	**0**	**0**	**0**	**1**	**0.1**	**3**	**4**	**0**	**0.4**	**1**
YGL189C	Rps26	0	0	0	0	3	0.2	0	3	0	0.2	0
**YLR167W**	**Rps31**	**0**	**0**	**0**	**0**	**1**	**0.1**	**2**	**2**	**0**	**0.3**	**1**
**YOL121C**	**Rps19**	**0**	**0**	**1**	**0**	**0**	**0.1**	**3**	**2**	**0**	**0.3**	**1**
YHL015W	Rps20	0	0	0	0	1	0.1	2	0	0	0.1	0
**60S**												
YGL031C	Rpl24	0	3	1	4	3	0.5	0	0	1	0.1	0
YDL136W	Rpl35	0	3	2	1	1	0.2	0	2	0	0.1	0
YHR010W	Rpl27	0	0	0	3	3	0.3	2	0	0	0.1	0
YGL103W	Rpl28	2	0	3	2	0	0.3	0	1	0	0,1	0
YBR191W	Rpl21	0	1	0	3	1	0.2	3	0	0	0.2	0
**YGL135W**	**Rpl1**	**0**	**0**	**1**	**1**	**1**	**0.2**	**4**	**1**	**0**	**0.3**	**1**
**YBL087C**	**Rpl25**	**0**	**0**	**2**	**1**	**0**	**0.2**	**3**	**2**	**0**	**0.3**	**1**
YBL092W	Rpl32	0	1	2	1	0	0.2	0	2	0	0.1	0
YGR085C	Rpl11	0	0	1	0	1	0.1	4	1	0	0.3	1
YHL033C	Rpl8	0	0	0	1	1	0.1	3	2	2	0.4	1
YIL052C	Rpl34	0	0	2	1	0	0.2	0	2	0	0.1	0

Gene Name	Protein Name	Brr2 control 1	Brr2 control 2	Rrp5 No RNase 1	Rrp5 No RNase 2	Rrp5 No RNase 3	Rrp5 No RNase % total peptides	Rrp5 RNase 1	Rrp5 RNase 2	Rrp5 RNase 3	Rrp5 RNase % total peptides	Selection
**YGL030W**	**Rpl30**	**0**	**0**	**0**	**1**	**0**	**0.1**	**3**	**1**	**0**	**0.3**	**1**
**YPL143W**	**Rpl33**	**1**	**0**	**0**	**1**	**0**	**0.1**	**1**	**1**	**0**	**0.1**	**1**
YDL075W	Rpl31	0	0	2	0	1	0.2	0	0	0	0	0
**Other proteins**												
YER165W	Pab1	0	4	12	7	23	2.4	3	9	0	0.8	0
**YOR341W**	**Rpa190**	**0**	**0**	**0**	**0**	**18**	**1.0**	**16**	**10**	**1**	**1.8**	**1**
YGL173C	Kem1	0	0	0	0	22	1.3	2	3	0	0.3	0
**YPR010C**	**Rpa135**	**0**	**0**	**0**	**0**	**7**	**0.4**	**9**	**3**	**0**	**0.8**	**1**
**YOR204W**	**Ded1**	**0**	**0**	**0**	**0**	**10**	**0.6**	**3**	**4**	**0**	**0.4**	**1**
YGR145W	Enp2	0	0	0	2	2	0.2	1	0	0	0.1	0
**YAL005C**	**Ssa1**	**1**	**1**	**8**	**10**	**7**	**1.4**	**13**	**2**	**0**	**1.0**	**1**
YNL209W	Ssb2	0	5	1	0	27	1.6	0	13	0	0.8	0
YLR259C	Hsp60	11	0	1	5	17	1.3	9	0	0	0.6	0
YDL229W	Ssb1	2	0	6	12	1	1.1	13	0	0	0.8	0
YNL064C	Ydj1	3	0	0	1	14	0.9	0	0	0	0	0
**YGR285C**	**Zuo1**	**0**	**0**	**0**	**0**	**9**	**0.5**	**3**	**1**	**0**	**0.3**	**1**
**YLR249W**	**Yef3**	**0**	**0**	**0**	**0**	**34**	**2.0**	**33**	**6**	**0**	**2.5**	**1**
**YOR133W**	**Eft1**	**0**	**4**	**0**	**0**	**39**	**2.2**	**22**	**6**	**0**	**1.8**	**1**
**YPR080W**	**Tef1**	**3**	**0**	**1**	**3**	**22**	**1.5**	**22**	**11**	**5**	**2.4**	**1**
YBR079C	Rpg1	0	0	0	0	13	0.7	4	2	0	0.4	1
YKL081W	Tef4	0	0	0	0	6	0.3	3	0	0	0.2	0
**YMR309C**	**Nip1**	**0**	**0**	**0**	**0**	**3**	**0.2**	**2**	**1**	**0**	**0.2**	**1**
YLR150W	Stm1	0	0	1	0	8	0.5	0	2	0	0.2	0
YGR162W	Tif4631	0	0	0	0	4	0.2	1	0	0	0.1	0
**YDL126C**	**Cdc48**	**0**	**0**	**0**	**2**	**10**	**0.7**	**11**	**8**	**0**	**1.2**	**1**
YJL080C	Scp160	0	0	0	0	23	1.3	4	0	0	0.3	0
**YDL145C**	**Cop1**	**0**	**0**	**0**	**0**	**3**	**0.2**	**4**	**4**	**0**	**0.5**	**1**
**YPL226W**	**New1**	**0**	**0**	**0**	**0**	**5**	**0.3**	**7**	**1**	**0**	**0.5**	**1**
YJL034W	Kar2	0	1	0	1	4	0.3	2	0	0	0.1	0
YDR238C	Sec26	0	0	0	0	2	0.1	1	1	0	0.1	1
YNL287W	Sec21	0	0	0	0	0	0	1	1	0	0.1	0
YDR381W	Yra1	0	0	0	0	4	0.2	0	2	0	0.1	0

Table S1

The table shows numbers of peptides identified for each protein listed. Three independent analyses are reported for Rrp5-HTP with (RNase) and without (no RNase) RNase treatment, and two for Brr2-HTP. Proteins indicated in bold showed recovery of Rrp5-associated peptides after RNase treatment in at least two of the three samples, that was at least 30% of the recovery without RNase treatment indicative of direct interaction, and at least 3 fold greater than recovery in Brr2 samples without RNase (except for the snoRNP proteins Nop1, Nop56 and Nop58, which are expected to be associated with intron-encoded snoRNAs and may therefore be associated directly with Brr2).

Supplementary Table S2. Strains used in this study.

Name	Description	Genotype	Source
BY4741	Wild-type	*MATa; his3Δ1*; *leu2Δ0*; *met15Δ0*; *ura3Δ0*	Reference strain
ySLD9	GAL::3HA-Rrp5	*MATa; his3Δ1; leu2Δ0; met15Δ0; ura3Δ0; GAL1::3HA::RRP5; Kan^R*	This Study
ySLD30	ySLD9 + (PTH-NTD, untagged CTD)	*MATa; his3Δ1; met15Δ0; GAL1::3HA::RRP5; pSLD3; pSLD6; ; Kan^R; Leu+, Ura+*	This Study
ySLD31	ySLD9 + (untagged NTD, PTH-CTD)	*MATa; his3Δ1; met15Δ0; GAL1::3HA::RRP5; pSLD4; pSLD5; ; Kan^R; Leu+, Ura+*	This Study
2D/483	Wild-type 2	MATα; *ura3-52; leu2-3,112; his3Δ200; lys2Δ201; trp1Δ; GAL2*	Reference strain
ASY057	Mrd1-HTP	MATα; *ura3-52; leu2-3,112; his3Δ200; lys2Δ201; GAL2; Mrd1-HTP-TRP1*	(Segerstolpe et al., 2013)
ASY079	Rrp5-HTP	MATα; *ura3-52; leu2-3,112; his3Δ200; lys2Δ201; GAL2; RRP5-HTP-TRP1*	This study
ASY088	GAL::3HA-Rrp5	MATα; *ura3-52; leu2-3,112; lys2Δ201; GAL2: HisMX6-PGAL1-3HA-Rrp5*	This study
ASY113	ASY088 + (PTH-NTD, untagged CTD)	MATα; *ura3-52; leu2-3,112; lys2Δ201; GAL2: HisMX6-PGAL1-3HA-Rrp5; pSLD3; pSLD6.*	This study
ASY114	ASY088 + (untagged NTD, PTH-CTD)	MATα; *ura3-52; leu2-3,112; lys2Δ201; GAL2: HisMX6-PGAL1-3HA-Rrp5; pSLD4; pSLD5.*	This study
ASY124	Arx1-HTP	MATα; *ura3-52; leu2-3,112; his3Δ200; lys2Δ201; GAL2; ARX1-HTP-TRP1*	This study
ASY125	Nop7-HTP	MATα; *ura3-52; leu2-3,112; his3Δ200; lys2Δ201; GAL2; ARX1-HTP-TRP1*	This study
ASY127	GAL::Rat1, ΔXrn1	MATα*; leu2-3,112; his3Δ200; lys2Δ201; GAL2; ΔXRN1::URA3; GAL1-Rat1-KanMX6*	This study
ySLD131	Nop12-GFP; Rrp5-HTP	ATCC 201388 MATa *leu2D0 met15D0*	This study
ySLD132	Utp10-GFP; Rrp5-HTP	ATCC 201388 MATa *leu2D0 met15D0*	This study
ySLD133	Utp20-GFP; Rrp5-HTP	ATCC 201388 MATa *leu2D0 met15D0*	This study
ySLD134	Utp21-GFP; Rrp5-HTP	ATCC 201388 MATa *leu2D0 met15D0*	This study
ySLD135	Nop58-GFP; Rrp5-HTP	ATCC 201388 MATa *leu2D0 met15D0*	This study
ySLD136	Kre33-GFP; Rrp5-HTP	ATCC 201388 MATa *leu2D0 met15D0*	This study
ySLD137	Rrp12-GFP; Rrp5-HTP	ATCC 201388 MATa *leu2D0 met15D0*	This study
ySLD138	Prp43-GFP; Rrp5-HTP	ATCC 201388 MATa *leu2D0 met15D0*	This study
ySLD139	Urb1-GFP; Rrp5-HTP	ATCC 201388 MATa *leu2D0 met15D0*	This study

Supplementary Table S3. Oligonucleotides used in this study.

Definition	Sequence (5'-3')
F5-RRP5	GAGTTTATTGTCACTACATTGGCCAGTCATTCAATGCCCGGAATTCGAGCTCGTTTAAAC
R5-RRP5	GTGGAAAATCTTCATCTCTCTTTCTTTTGGTGGAAGCTACCATGCACTGAGCAGCGTAATCTG
NTD-RRP5 XmaIO1	GGGGGCCCGGGGGATGGTAGCTTCCACCAAAAGAAAGAGAGATGAAGATTTTCC
NTD-RRP5 XmaIO2	GGGGGCCCGGGTTATTCCTTCAATGTTAGGGAGAAATCATTTAACGCATCCG
CTD-RRP5 XmaIO1	GGGGGCCCGGGGGATGTTTGAAGACAAAATTAACAACGTCATTCCAACTACCG
CTD-RRP5 XmaIO2	CCCCCCCCGGGTTATTCGTCTGCTTTTTGAGATTCATGGCTAGCGACATACTC
Rrp5-HTP Fw	CTAAAGCTACTGAGTATGTCGCTAGCCATGAATCTCAAAAAGCAGACGAAGAGCACCATCACCATCACC
Rrp5-HTP Rev	TTACAAACTTAGCCATTTATATTACTTTACAGTTAAAAATCCATCAGGAATACGACTCACTATAGGG
Nop7-HTP Fw	CAAACAAAAAGCTAAACTGAATAAACTAGATTCCAAGAAAGAGCACCATCACCATCACC
Nop7-HTP Rev	AGACAAAATTTTTGAGAGGCTATTGGAAAAGAAGAGAAAATACGACTCACTATAGGG
Arx1-HTP Fw	TGAGACATCAAATGGCGGAGTTGAAGAAACCATGAAAATGGAGCACCATCACCATCACC
Arx1-HTP Rev	TATATTATTTATATACTAGCTTTAGAAATGATGAAGTTTCTACGACTCACTATAGGG
Xrn1 F1	ACTTGTAACAACAGCAGCAACAAATATATATCAGTACGGTCCAGCTGAAGCTTCGTACGC
Xrn1 R1	TAAAGTAACCTCGAATATACTTCGTTTTTAGTCGTATGTTGCATAGGCCACTAGTGGATC
Rat1 F5	GGTTCTTCAAGGAAGTGCTACAGCCAAGTTTCGTAATATAGAATTCGAGCTCGTTTAAAC
Rat1 R5	GATATTTTCGAGATAGCCATCTGAAAAATGACGGAACACCGCACTGAGCAGCGTAATCTG
Probe 1	CATGGCTTAATCTTTGAGAC
Probe 2	GGCCAGCAATTTCAAGTTA
Probe 3	CTCCGCTTATTGATATGC
Probe 4	GCTCTCATGCTCTTGCC
Probe 5	CCAGTTACGAAAATTCTTG
NME1	GCAATAGAGGTACCAGGTCAAGAAG

Table S4: Summary of Rrp5 CRAC experiments.

Constructions	Number of Repeats
Rrp5-HTP Full-length *in vitro*	2
Rrp5-HTP Full-length *in vivo*	3
PTH-Rrp5 NTD *in vivo*	3
PTH-Rrp5 CTD *in vivo*	3

Data used to make figures are available on GEO (GSE47680)

SUPPLEMENTARY INFORMATION

EXTENDED EXPERIMENTAL PROCEDURES

Yeast strains.

Strains used are listed in Table S2. For the CRAC approach, the genomic copy of Rrp5 was C-terminally tagged (Longtine et al., 1998) for expression as a Rrp5-His6-TEV-2ProteinA (HTP) fusion in the BY4741 (MATa; *his3Δ1*; *leu2Δ0*; *met15Δ0*; *ura3Δ0*) background. For analysis of NTD or CTD domains by CRAC, the *RRP5* promoter was exchanged for a P_{GAL} promoter, allowing construction of YSLD9 strain (BY4741, MATa; *his3Δ1*; *leu2Δ0*; *met15Δ0*; *ura3Δ0*, P_{GAL}*::3HA-RRP5*, KanR). NTD and CTD regions of Rrp5 were inserted into XmaI site of pRS415-nPTH expression vectors allowing expression of a N-terminally tagged, generating pSLD3 and pSLD4 respectively. The NTD and CTD cDNAs were also cloned into XmaI site of plasmid pRS416 allowing expression of untagged moiety to complement depletion of the *RRP5* genomic copy, generating pSLD5 and pSLD6 respectively. Genome-encoded *RRP5*, *ARX1* and *NOP7* expressing fusions with a C-terminal His6-TEV-Protein A (HTP) tag were created using a one-step PCR strategy, with amplification of the HTP tag from the pBS1479/HTP vector (Granneman et al., 2009). The HTP tags did not detectably affect cell growth. Oligonucleotides used are listed in Table S2.

Immunoprecipitation and RNA analysis.

Extracts from 220 OD_{600} units of exponential growing cells were used for affinity purification. For strain ASY113 and ASY114, expression of endogenous Rrp5 were shut off in glucose for 12 hours before harvest. Briefly, cell pellets were lysed by vortexing the cells with Zirconia beads in the presence of 1 ml lysis buffer (50 mM Tris-HCl (pH 7.8), 1.5 mM MgCl2, 150 mM NaCl, 0.1% Nonidet P-40, and 5 mM β-mercaptoethanol, 1X Complete Protease Inhibitor cocktail EDTA free (Roche) 20 mM Vanadyl Ribonucleoside complex and 0.1U μl-1 of RNaseOUT (Invitrogen)). The extracts were cleared by centrifugation and the supernatants were incubated for 2 h with IgG sepharose beads (GE-healthcare). The IgG beads were washed 3x5 minutes with 10 ml of TMN150 buffer (50 mM Tris-HCl (pH 7.8), 1.5 mM MgCl2, 150 mM NaCl, 0.1% Nonidet P-40, and 5 mM β-mercaptoethanol). Bound complexes were eluted with GST-tobacco etch virus (TEV) protease and EDTA and SDS were added to a final concentration of 10mM and 1% respectively. The RNA was phenol/chloroform purified and ethanol precipitated.

Protein crosslinking and immunoprecipitation

Rrp5-binding proteins were initially identified by formaldehyde crosslinking. Cultures expressing HTP-Rrp5 were grown and harvested; cells were disrupted and extracted with PSN [PBS (Phosphate Buffered Saline), 2mM $MgCl_2$, 0.4% NP40, Protease Inhibitors (Roche), RNAsine (Promega)] buffer and clarified by centrifugation as described for CRAC technique. Cell extracts were incubated for 15 min with IgG sepharose and washed twice with PSN, twice with PSN300 (PNS + 0.3M NaCl) and twice with PBS. Protein complexes on sepharose were crosslinked in 0.1% formaldehyde in PBS for 3 min at room temperature. For assessment of RNA-independent interactions, protein complexes were washed with TMN150 and treated with 1:100 dilution of RNace-It Cocktail (Stratagene) for 10 min prior to formaldehyde crosslinking. Following crosslinking the reaction was quenched by addition of Glycine and Tris-HCl to final concentrations of 0.15 M each for 5 min. Complexes on sepharose were washed extensively with 8M Urea in TMN150 and then PBS. After brief (3 min) de-crosslinking in NuPage sample buffer with 0.1M DTT and 1% SDS at 95°C samples, proteins were separated on NuPage gel and protein bands were visualized by Imperial Protein Stain (Thermo Scientific). Despite the partial de-crosslinking needed to release Rrp5-HTP from the column, complexes were observed to migrate more slowly than 191 kDa (the size of Rrp5). These were excised for analysis by mass spectrometry.

Protein interactions with Rrp5 found by mass spectrometry analysis were confirmed by immunoprecipitation. To do so, we transformed GFP-tagged strains from the Invitrogen collection, to obtain Protein X-GFP; Rrp5-HTP strains. Cultures of these strains were grown and harvested. Cells were disrupted and proteins extracted with TMN500 (50mM Tris-Hcl pH= 7.5, 500mM NaCl, 2mM MgCl2, 0.1% Triton, 1mM DTT, Protease inhibitor). For each extract, 12mg equivalent of total protein extract was used per IP. To identify RNA-independent interactions, a 1:100 dilution of RNace-it Cocktail (Stratagene) was added for 10 min at 22°C. To remove the Protein A-tag from Rrp5-HTP, extracts were incubated with 40u of TEV protease for 2 h at 18°C. To eliminate any remaining Protein A tag and reduce background, extracts were incubated with 50 μl of IgG-sepharose beads for 1 h. Protein A-free supernatants were then incubated with 25 μl anti-GFP nanobody-agarose beads (Chromotek) for 1.5 h. Beads were washed 3 times with TMN500 and proteins were eluted from the column by addition of 2X SDS loading buffer. Samples were boiled for 10min before loading onto gels for western-blotting.

Primer extension.

Affinity purified RNA and 1% of input RNA from untagged or HTP tagged strains were used for primer extensions. The RNA was incubated at 80°C for 5 minutes and then hybridized with γP^{32} labeled probes (probes listed in Supplementary Table S2) for 90 min at 50°C. Superscript III reverse transcriptase (Invitrogen) was used for reverse-transcription. The cDNA was separated on a 6% polyacrylamide gel, dried and signals were detected with a Fuji FLA3000 scanner.

Sucrose gradients.

200 ml of exponentially growing yeast cells were incubated with 0.1 mg ml^{-1} of cycloheximide (Sigma, St. Louis) at 30°C for 10 min. The cells were washed in water and lyzed with zirconia beads in lysis buffer (20 mM HEPES, pH 7.5, 10 mM KCl, 2.5 mM Mg_2Cl, I mM EGTA, pH8.0; 0.1 mg^{-1} cycloheximide, 1 mM DTT). The extract was centrifuged at 15,000 g for 10 min and 100 OD_{260nm} units of the supernatant was layered onto a 10-50% sucrose gradient in lysis buffer, lacking cycloheximide and DTT. The gradients were centrifuged in a SW40 rotor (Beckman) at 39,500 rpm for 155 min at 4°C. 500 μl fractions were collected. RNA was extracted from each fraction and analyzed by northern blot.

Northern-blot analysis.

RNA was separated in 1% agarose-formaldehyde gels and transferred to Zeta-probe membrane (Bio-Rad, Hercules, California, USA). Oligonucleotide probes were labeled with T4 polynucleotide kinase (New England Biolabs, Ipswich, Massachusetts USA). Prehybridization and hybridization were performed at Tm-9°C in 5xSSPE (1xSSPE is 150 mM NaCl, 10 mM Na_2HPO_4 H_2O, 1 mM EDTA, ph 7.4), containing 7% SDS, 1x Denhart solution and 100 μg/ml salmon sperm DNA. The filters were washed in 3xSSPE, 0.5% SDS at hybridization temperature and in 1xSSPE, 0.1%SDS at 37°C. A ^{32}P-labelled RNA hybridization probe for NME1 was made by in vitro transcribing an NME1 specific PCR product with a built in T7 promoter. The RNA probe was hybridized in 50% formamide, 1.5x SSPE containing 1% SDS, 0.5% Blotto, 100 μg ml^{-1} salmon sperm DNA. The filters were washed in 2xSSC, 0.1% SDS at room temperature, 1xSSC, 0.1% SDS at 50°C and 0.1xSSC, 0.1% SDS at 50°C for 15 min each. Hybridization signals were analyzed with a Fuji FLA3000 scanner.

SUPPLEMENTARY REFERENCES

Bradatsch, B., Leidig, C., Granneman, S., Gnadig, M., Tollervey, D., Bottcher, B., Beckmann, R. and Hurt, E. (2012) Structure of the pre-60S ribosomal subunit with nuclear export factor Arx1 bound at the exit tunnel. *Nat Struct Mol Biol*, **19**, 1234-1241.

Granneman, S., Petfalski, E. and Tollervey, D. (2011) A cluster of ribosome synthesis factors regulate pre-rRNA folding and 5.8S rRNA maturation by the Rat1 exonuclease. *Embo J*, **30**, 4006-4019.

Segerstolpe, A., Granneman, S., Bjork, P., de Lima Alves, F., Rappsilber, J., Andersson, C., Hogbom, M., Tollervey, D. and Wieslander, L. (2013) Multiple RNA interactions position Mrd1 at the site of the small subunit pseudoknot within the 90S pre-ribosome. *Nucleic Acids Res*, **41**, 1178-1190.
